# Relationships of pelagic ciliates with the microbial food web components at a coastal station in the oligotrophic Eastern Mediterranean Sea: temporal and vertical variability

**DOI:** 10.1093/plankt/fbab053

**Published:** 2021-08-27

**Authors:** Filomena Romano, Paraskevi Pitta

**Affiliations:** Marine Biological Section, University of Copenhagen, Helsingør DK-3000, Denmark; Institute of Oceanography, Hellenic Centre for Marine Research, Former US Base at Gournes, 71500 Heraklion, Crete, Greece; Marine Biological Section, University of Copenhagen, Helsingør DK-3000, Denmark

**Keywords:** mixotrophic ciliates, microbial food web, potential prey, biomass dynamics, potential prey–predator correlations

## Abstract

The annual/temporal and vertical dynamics of the microbial food web (MFW) was studied in a coastal station of the oligotrophic Eastern Mediterranean Sea. The present study analyzed the changes of all components of the MFW with a specific focus on the relationships between different size classes of heterotrophic and mixotrophic ciliates with their potential prey. The MFW was dominated by heterotrophic picoplankton in all months and depths analyzed, whereas autotrophic nanoplankton took advantage in cold months with higher nutrient availability. On the other hand, mixotrophic microplankton biomass was higher in summer when nutrients and chlorophyll-a were scarce.

As part of the mixotrophic biomass, mixotrophic ciliates were correlated with their “potential” prey at the surface and deep chlorophyll maximum. Large mixotrophic ciliates (*L. strobila*) were more selective in terms of potential prey, showing a correlation with *Synechococcus*. On the other hand, mixotrophic nanociliates (*Strombidium dalum*) were correlated differently with different potential prey according to depth, supporting the idea that nanociliates could be more generalists in terms of prey selection. Because the relationships between mixotrophic ciliates and their potential prey are still poorly studied, this work represents the start for further investigation.

## INTRODUCTION

Marine pelagic microbial food web (MFW) comprises organisms of different size classes from fempto- to microplankton belonging to different plankton groups from viruses to dinoflagellates and ciliates (Azam *et al.,* 1983; Pomeroy, 1974; Sherr and Sherr, 1988). These organisms are characterized by different trophic modes such as photo-autotrophy, phago-heterotrophy and mixotrophy, and are interconnected with complex trophic relationships that depend on size and are further complicated by mixotrophy prevailing in most of these plankton groups. However, plankton organisms also depend on environmental variables that vary with time/season and depth. Therefore, in order to understand the pelagic system function and dynamics, it is crucial to study not only the food web structure (by studying all components and their complex relationships) at specific time points but also the temporal variability of these components.

MFW structure is usually described based on different characteristics such as trophic modes and size. Autotrophic prokaryotes like *Synechococcus* and *Prochlorococcus* as well as pigmented picoeukaryotes are all part of picophytoplankton (Van Dongen-Vogels *et al.,* 2012). However, when heterotrophic bacteria are also considered, one may talk about picoplankton community structure (Otero-Ferrer *et al.,* 2018; Zubkov *et al.,* 1998; Zubkov *et al.,* 2000). Because picoplankton was traditionally easier counted and analyzed, compared to larger plankton groups, first by epifluorescence microscopy and more recently by flow cytometry, the relative contribution of the picoplankton groups to the MFW has been thoroughly studied in the marine environment (Agawin *et al.,* 1998; Azam *et al.,* 1983; Caroppo, 2000; Caroppo *et al.,* 2006; Hall and Vincent, 1994; Mackey *et al.,* 2002; Modigh *et al.,* 1996; Partensky *et al.,* 1999). In contrast, other groups such as nanoplankton and microplankton are still much less documented.

One of the most frequent ways to describe the structure of the marine MFW is to establish numerical relationships between different groups with respect to their abundance or biomass. For example, the relationship between heterotrophic bacteria and heterotrophic nanoflagellate abundance was studied by Sanders *et al.* (1992)) and Gasol *et al.* (1997) by compiling data collected from many ecosystems. Similarly, Fenchel (2008)) stated that bacteria and flagellates in the water column are around 10^6^ and 10^3^ cells mL^−1^, respectively.

Advances in the field of marine microbial ecology have increased with the introduction and study of the carbon and energy flow through the marine MFW. On average, ~50% of the organic carbon produced by phytoplankton, the main marine primary producers, is channeled through the so-called “microbial loop” (Whitman *et al.,* 1998).

Despite the fact that picoplankton has received a lot of attention in marine ecological studies, the term marine food web structure was introduced only when flagellates and ciliates were also considered in these studies and incorporated into the microbial community (Garrison *et al.,* 2000).

Ciliates are an important component of the pelagic ecosystem, as they represent the link between the MFW on one hand and mesozooplankton and the higher trophic levels on the other. Especially in oligotrophic environments (Atlantic Ocean and Mediterranean Sea), ciliates are even more important because they are the main grazers due to the small size of primary producers (Burkill *et al.,* 1993; Pitta and Giannakourou, 2000; Sherr and Sherr, 1987). Pelagic planktonic ciliates play different roles in the marine MFW according to their different trophic modes and sizes; mixotrophic and heterotrophic ciliates, on one hand, and nano- and microciliates on the other, have different temporal and vertical distributions in the oligotrophic Mediterranean Sea (Bojanić *et al.,* 2012; Dolan *et al.,* 2019; El-Shabrawy *et al.,* 2018; Heneash *et al.,* 2015; Pitta and Giannakourou, 2000; Romano *et al.,* 2021). The biomass of the mixotrophic and heterotrophic ciliates as part of the MFW, as well as the fluxes of carbon and energy mediated by them, have been determined for a variety of marine systems, ranging from coastal to open ocean environments (Trombetta *et al.,* 2020; Yang *et al.,* 2021; Fuhrman, 1999). However, the interaction between different size classes and trophic modes of ciliates and the other components of the MFW is still poorly studied in the ultra-oligotrophic environments (Burkill *et al.,* 1993; Capriulo *et al.,* 1991; Sherr and Sherr, 1987). More specifically, to our knowledge, very little is known on the vertical and temporal variability of the potential impact and interaction of different size classes of mixotrophic and heterotrophic ciliates with the other components of pico- and nanoplankton.

Therefore, the objective of this paper was to assess the temporal and vertical dynamics of the biomass of all components of the MFW with a specific focus on the role of ciliates in the structure and function of the food web. More specifically, we measured the biomass of all different components of the MFW (from bacteria to ciliates) in samples collected in a monthly basis throughout 1 year from the euphotic zone (surface to 120 m depth) at a coastal station of the Eastern Mediterranean Sea in order to identify: (i) the seasonal and vertical variation of the MFW structure in this coastal station; (ii) the dynamics of the pico-, nano- and microplankton as well as the contribution of autotrophic, mixotrophic and heterotrophic biomass to each group of organisms; and (iii) the correlation between different groups of pelagic ciliates with both abiotic (temperature, salinity and nutrients) and biotic (biomasses of the other components of the MFW) variables at the surface and deep chlorophyll maximum (DCM).

Our hypotheses were: (i) biomass variation trends of different components of the MFW should be time/season and depth dependent; (ii) in this oligotrophic system, picoplankton and nanoplankton should be heterotrophic and autotrophic, respectively but, in contrast, microplankton is expected to be mixotrophic, taking into account that the most abundant components are dinoflagellates and ciliates; and (iii) the potential impact of mixotrophic and heterotrophic ciliates of different sizes on their potential prey should be related to different environmental and biological factors.

## METHOD

### Sampling, abiotic variables and chlorophyll-a

The coastal station POSEIDON-HCB (Heraklion Coastal Buoy, 35.426°N–25.072°E, max depth 180 m) at Heraklion Bay, Cretan Sea, Greece was sampled on a monthly basis from January to December 2019 (Fig. S1). A Seabird CTD profiler was used for the profiles of the water column (temperature, salinity). Using 5 L Niskin bottles, samples were collected at the euphotic zone, specifically at 2, 10, 20, 50, 75, 100 and 120 m. Because of adverse sea conditions, sampling was not performed in February and August.

Analyses of dissolved nutrients and chlorophyll-a (Chl a) were performed directly after sampling. Phosphate concentration was measured as described by Rimmelin and Moutin (2005), nitrate, nitrite and silicate as described in Strickland and Parsons (1972) and ammonium according to Ivančič and Degobbis (1984). Detection limits for phosphate, nitrate, nitrite, silicate and ammonium were 0.018, 0.017, 0.010, 0.025 and 0.019 μM, respectively. In the present study, NO_2_, NO_3_ and NH_4_ were summed and presented as DIN. Water samples for Chl a were filtered through 0.2 μm polycarbonate membranes using a vacuum filtration system. The extraction of pigments was performed in 90% acetone and, after 24 hours, the Chl a concentration was detected by means of a fluorometer according to Yentsch and Menzel (1963).

### Picoplankton

Subsamples of 2 mL of water were fixed with 0.2 μm prefiltered glutaraldehyde (final concentration 2% v/v) and processed for heterotrophic bacteria and Archaea (Bact) counts according to Marie *et al.* (1999). After fixation, samples were kept at room temperature for 15 minutes and then, frozen in liquid nitrogen and stored at −80°C until further analysis that was carried out within 6 months. The analysis was performed using a FACSCalibur™ flow cytometer (Becton Dickinson) equipped with a 488-nm argon laser. The nucleic acid stain SYBR Green I (Molecular Probes, USA) was used for heterotrophic bacteria analysis (Bact), at 4× final concentration. Samples were first 1:10 diluted with Tris-EDTA buffer, then stained with SYBR Green I and incubated for 20 minutes in the dark. Heterotrophic bacteria were distinguished based on their green fluorescence due to the staining and scatter properties. Cyanobacteria *Synechococcus* (Syn) and *Prochlorococcus* (Proc) as well as autotrophic picoeukaryotes (pEuk) were counted without fixation and staining steps, using their characteristic auto-fluorescence chlrorophyll/phycoerythrin signals and side scatter. Biomass of *Synechococcus*, *Prochlorococcus*, picoeukaryotes and heterotrophic bacteria was calculated by using the following conversion factors: 250 fg C cell^−1^ and 60 fg C cell^−1^ as in Li *et al.* (1992), 1500 fg C cell^−1^ (Zubkov *et al.,* 1998) and 20 fg C cell^−1^ (Lee and Fuhrman, 1987).

### Nanoplankton

For the enumeration of pigmented and nonpigmented nanoflagellates (PnFlag and NPnFlag, respectively), 20 mL of subsample were fixed with 2% formaldehyde (buffered with sodium tetraborate decahydrate and filtered for particle removal through 0.45 μm membranes). Fixed samples were stored in the dark at 4°C for 30 minutes. Samples were then concentrated to ca. 10 mL on 0.6 μm black polycarbonate membranes of 25 mm diameter, stained with 0.2 mg L^−1^ DAPI (4,6 diamino-2-phenylindole) for 10 minutes and finally collected on the membranes (Porter and Feig, 1980), which were mounted on slides and subsequently stored at −20°C. The analysis was carried out within 3 weeks after fixation. The enumeration of pigmented and nonpigmented nanoflagellates was performed using epifluorescence microscopy under ultraviolet and blue excitation. Pigmented nanoflagellates were distinguished from the nonpigmented ones by the emission of red fluorescence due to the presence of their own chloroplasts, on three transects. Nanoflagellates were counted in size classes and the sphere shape was assumed to calculate the biovolume. Biomass was calculated using the conversion factor 183 fg C μm^−3^ (Caron *et al.,* 1995).

Biomass of dinoflagellates and ciliates <20 μm was also considered as nanoplankton biomass and was added to the one of nanoflagellates.

### Microplankton

Samples for microplankton groups were fixed with 2% of acid Lugol’s solution and stored in dark bottles at 4°C. Analyses were performed within 1 month after each sampling. Subsamples of 100–250 mL were settled for ~24 hours in Utermöhl chambers (Utermöhl, 1931) and the enumeration of diatoms (Diat), dinoflagellates (Dino) and ciliates (Cil) was performed with an inverted microscope at 150× magnification using bright field. Diatoms and larger dinoflagellates and ciliates (>30 μm in length) were identified at the species level when possible; especially for ciliates, this was done following Laval-Peuto and Rassoulzadegan (1988), Lynn *et al*. (1988), Montagnes *et al*. (1988), Lynn and Montagnes (1991), whereas <30 μm dinoflagellates and ciliates were identified at the genus level following Halse *et al*. 1996.

For diatoms, dinoflagellates and ciliates, the length and width of several specimens were measured to estimate biovolume, assuming geometric shapes, then the C content cell^−1^ for each species was calculated using conversion factors pg C cell^−1^ = 0.288 × volume^0.811^ for diatoms; pg C cell^−1^ = 0.760 × volume^0.819^ for dinoflagellates (Menden-Deuer and Lessard, 2000); pg C cell^−1^ = (volume × 0.053) + 444.5 for tintinnids (Verity and Langdon, 1984); pg C cell^−1^ = volume × 0.19 for other ciliates (Putt and Stoecker, 1989). Finally, biomass was calculated for all groups according to different size classes and trophic modes.

Dinoflagellates were divided into four size classes according to their cell length (<20, 20–50, 50–180 and > 180 μm), according to Le Bescot *et al.* (2016) and the trophic mode of specimens with assigned species names was assessed from the literature (Matishov *et al.,* 2000).

Ciliates were divided into two different size classes according to whether they were smaller or >20 μm, in order to place them in nanoplankton (denoted as nanociliates) or microplankton (denoted as microciliates), respectively. For the trophic mode assignment, specimens with assigned species names were recognized as mixotrophic or heterotrophic, based on literature. For specimens not assigned to a species, the percentage of mixotrophic and heterotrophic cells was calculated based on epifluorescence counts as explained in Romano *et al.* (2021).

Afterward, ciliates were divided into four groups: mixotrophic nanociliates (MnCil), mixotrophic microciliates (MmCil), heterotrophic nanociliates (HnCil) and heterotrophic microciliates (HmCil).

Both nanodinoflagellates and nanociliates were considered as part of nanoplankton.

### Statistical analysis and alpha diversity

The integrated abundances and biomasses of all components of the MFW were calculated, whereas the annual average was calculated at each depth.

All analyses were conducted using vegan package of R language version 4.0.1. Pearson correlation analysis was used to estimate relationships between biological and environmental variables, and data were transformed using square root transformation. Distance matrix was performed based on environmental variables (temperature, salinity, DIN, SiO_4_, and PO_4_), and biomasses of all MFW components.

Alpha diversity was measured temporally (for each month) and vertically (at each depth) using Shannon’s H index, richness value and Pielou’s Evenness index according to ciliate abundance. For the temporal evaluation of the alpha diversity, integrated values were used, whereas for the vertical evaluation, values of each depth were averaged (annual average). Rank abundance analysis was also carried out with R language version 4.0.1 and the integrated dataset, transformed using square root transformation, was used for temporal ranks, whereas the annual average was used for vertical ranks.

The most abundant species for four ciliate groups were taken into account for a Pearson correlation matrix and a canonical correlation analysis (CCA) between their abundance and their potential prey (pico- and nanoplanktonic groups). Both datasets of ciliate abundance and potential prey abundance were transformed with log(X + 1) in order to avoid the problem with many 0 s. In all analyses conducted for ciliates, only specimens with assigned species names were taken into account.

## RESULTS

### Temporal and vertical variation of environmental variables

During 2019, the water column was mixed from January to April, and also in December and stratified from May to November (Fig. S8).

Temperature varied from 15.34°C in March to 26.59°C in July (Table S1). Salinity ranged between 38.74 and 39.38 psu, with the lowest salinity value observed in March and the highest one in September. Both the lowest and highest concentrations of chlorophyll a (Chl a) occurred in March, 0.00 and 0.79 μg L^−1^, respectively (Table S1). Over the year 2019, concentrations of DIN, SiO_4_ and PO_4_ were in general very low; the highest values were measured in March for all three nutrients, whereas the lowest concentrations were detected in July and October for DIN and SiO_4_, respectively, and were below detection limit for PO_4_ in January and April. DIN varied from 0.32 to 2.41 μM, PO_4_ from 0.00 to 0.09 μM, and SiO_4_ concentration from 0.33 to 2.32 μM. Regarding vertical distribution, the highest DIN concentration was at 10 m depth in March, but at 120 m depth for all the other months (Fig. S2). Same situation was found for SiO_4_, where the highest concentration was at 2 m depth during March, but for all the other months, below the DCM. PO_4_ concentrations, on the other hand, were always very low except for 10 m depth in March and 100 m depth for the rest of the months (Fig. S2).

### Temporal and vertical variation of MFW components

**Fig. 1:**
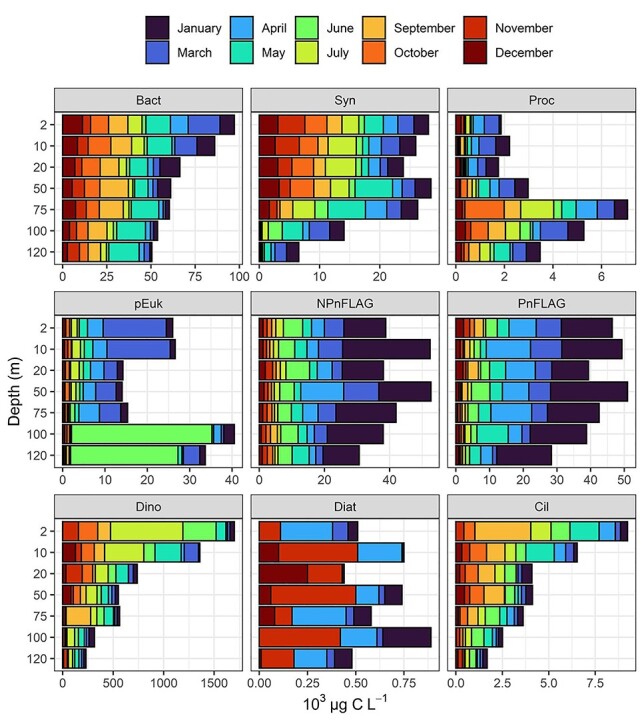
Vertical distribution of biomass of heterotrophic bacteria (Bact), *Synechococcus* (Syn), *Prochlorococcus* (Proc), picoeukaryotes (pEuk), nonpigmented nanoflagellates (NPnFLAG), pigmented nano flagellates (PnFLAG), dinoflagellates (Dino), diatoms (Diat) and ciliates (Cil) at each month and depth sampled.

The MFW components were divided into three groups according to size: picoplankton, nanoplankton and microplankton. Picoplankton included prokaryotes with a size range of 0.2–2 μm (heterotrophic bacteria and Cyanobacteria *Synechococcus* and *Prochlorococcus*) and picoeukaryotes. Nanoplankton consisted of nanoflagellates (both pigmented and nonpigmented flagellates with a size range of 2–20 μm), and also comprised ciliates and dinoflagellates <20 μm. Microplankton contained dinoflagellates, diatoms and ciliates in the size range of 20–200 μm. Abundances and biomasses of the eight MFW components varied with month and depth (Table I, Fig. 1).

**Table I:** Integrated abundance (cells m^−2^) and biomass (μg C m^−2^) of heterotrophic bacteria (Bact), Cyanobacteria (Cyano), picoeukaryotes (pEuk), pigmented nanoflagellates (PnFlag), nonpigmented nanoflagellates (NPnFlag), diatoms (Diat), dinoflagellates (Dino) and ciliates (Cil) for all months sampled

	Month	Bact	Cyano	pEuk	PnFlag	NPnFlag	Diat	Dino	Cil
		10^10^	10^9^	10^8^	10^9^	10^9^	10^8^	10^6^	10^6^
Abundance	January	2958	2100	1205	2449	2415	187	4214	348
	March	1968	2377	3953	623	722	8	8267	285
	April	2044	1518	1834	1012	653	536	4094	348
	May	7270	2143	637	705	526	0	9158	305
	June	1152	1118	9781	352	675	0	9141	445
	July	2323	2007	494	185	176	0	16 968	399
	September	6210	1431	290	147	172	0	6185	523
	October	3944	1777	420	97	200	0	6511	364
	November	3367	789	259	112	206	169	4477	198
	December	2576	941	271	127	139	97	3475	256
		10^3^	10^3^	10^3^	10^3^	10^3^	10^3^	10^3^	10^3^
Biomass	January	592	161	122	1826	1851	12	34	39
	March	394	172	298	546	471	3	40	25
	April	409	117	185	494	765	24	20	32
	May	1454	181	64	398	533	0	96	51
	June	230	90	988	510	266	0	72	75
	July	465	152	50	133	140	0	144	43
	September	1242	103	29	130	111	0	86	93
	October	789	123	42	152	74	0	57	62
	November	673	67	26	156	85	40	56	29
	December	515	73	27	105	96	7	36	24

On average, the biomass contribution of heterotrophic bacteria, Cyanobacteria and picoeukaryotes to the whole picoplanktonic biomass was 59, 24 and 17%, respectively. Heterotrophic bacteria dominated picoplankton in terms of both abundance and biomass (Table I). Their integrated abundance and biomass were higher during May and September, whereas the lowest values were found in June. Heterotrophic bacteria range was 1152–7270 × 10^10^ cells m^−2^ and 230–1454 × 10^3^ μg C m^−2^, respectively, for integrated abundance and biomass. Regarding vertical distribution, heterotrophic bacteria biomass reached the highest value at 2 m in March, whereas the lowest density was at 50 m in June (Fig. 1).

**Fig. 2:**
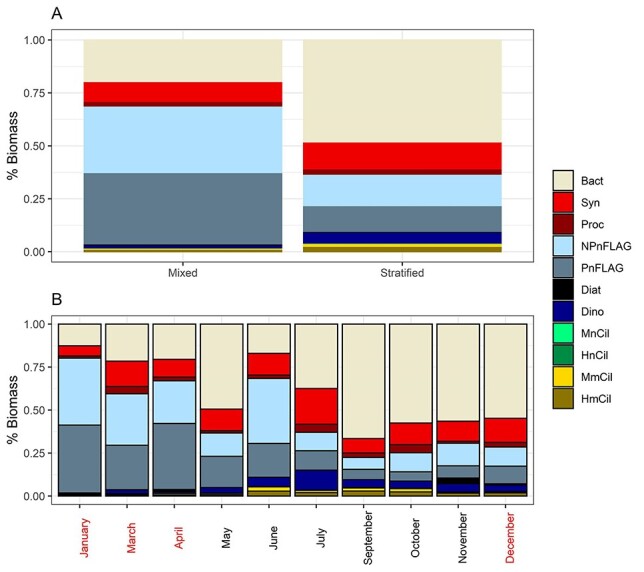
Relative biomass of all the components of the MFW for mixed and stratified water column. Months where the mixed water column occurred are inred.

Cyanobacteria integrated abundance was 10 times lower compared to heterotrophic bacteria (Table I); their abundance range was 789–2377 × 10^9^ cells m^−2^, whereas the biomass range was 67–181 × 10^3^ μg C m^−2^. The lowest biomass value was detected at 120 m in October while the highest one at 75 m in July (Fig. 2). Picoeukaryotes were more abundant during June and integrated abundance was in the range of 259–9781 × 10^8^ cells m^−2^ while integrated biomass in the range of 26–988 × 10^3^ μg C m^−2^ (Table I).

Nanoplankton biomass was almost equally distributed to nonpigmented nanoflagellates, nanodinoflagellates and pigmented nanoflagellates, whereas the contribution of nanociliates was much less (35.60, 32.24, 31.86 and 0.28%, respectively, data not shown). Total flagellate biomass was higher during January until May, and then it dropped significantly during summer (Table I, Fig. 1). The highest integrated abundance and biomass of both pigmented and nonpigmented nanoflagellates were found in January (2449 and 2415 × 10^9^ cells m^−2^, respectively, and 1826 and 1851 × 10^3^ μg C m^−2^, respectively); the lowest abundances for pigmented and nonpigmented nanoflagellates were in October and December, respectively (97 and 139 × 10^9^ cells m^−2^) and in December and October, respectively (105 and 97 × 10^3^ μg C m^−2^ for biomass, Table I). In terms of vertical distribution, pigmented and nonpigmented nanoflagellates showed the maximum biomass at 75 and 10 m in January, whereas the lowest biomass value was found at 120 m in September for both of them (Fig. 1). The integrated abundance ranged between 2243–10 956 × 10^6^ cells m^−2^ and 6.90–19.71 × 10^6^ cells m^−2^, respectively, for nanodinoflagellates and nanociliates (data not shown). In terms of vertical abundance distribution, nanodinoflagellates ranged between 267 (December 120 m) and 16 209 (July 2 m) cells L^−1^, whereas nanociliates ranged between 0 (December 100 m) and 440 (May 10 m) cells L^−1^.

For microplankton components, on average, diatoms were detected only during the mixing period and were not present or were present at very low densities during the stratification period (Table I), whereas dinoflagellates and ciliates were more abundant during the stratification period for both abundance and biomass. The diatom range was 0–536 × 10^8^ cells m^−2^ for integrated abundance and 0–40 × 10^3^ μg C m^−2^ for integrated biomass; the highest integrated abundance was detected in April while the highest biomass in November. Dinoflagellate and ciliate integrated abundance and biomass were very high during the stratification period. More specifically, dinoflagellates had a range of integrated abundance and biomass of 10^6^ × 3475 (December)—16 968 (July) cells m^−2^ and 10^3^ × 20 (April)–144 (July) μg C m^−2^, respectively. Ciliate integrated abundance ranged from 10^6^ × 198 (November) to 523 (September) cells m^−2^, whereas integrated biomass varied from 10^3^ × 24 (December) to 93 (September) μg C m^−2^ (Table I). Abundance of diatoms, dinoflagellates and ciliates fell in the range of 0–552, 980–31 280 and 80–1750 cells L^−1^, respectively (data not shown). The highest abundance of diatoms was at 75 m in April, whereas the highest one of dinoflagellates and ciliates was detected at 2 m in July and September, respectively (Fig. 1).

In terms of relative contribution of each plankton group to total integrated biomass, pigmented and nonpigmented nanoflagellates dominated during the mixing period for >70% of the total biomass, whereas heterotrophic bacteria dominated during the stratification one (Fig. 2A). The highest relative contribution of heterotrophic bacteria was found in September (Fig. 2B). January was the month with the highest relative contribution of total nanoflagellates (NPnFLAG and PnFLAG together), whereas dinoflagellates reached their highest relative biomass in July. Relative contribution of ciliates to total biomass was highest in June, July, September and October (Fig. 2B). More specifically, the ciliate biomass was dominated by HmCil and MmCil.

Annual average abundance and biomass of heterotrophic bacteria, dinoflagellates and ciliates showed a constant decrease with depth (Fig. S3). Cyanobacteria, nanoflagellates and diatoms, instead, did not decrease with depth but showed subsurface maxima of both abundance and biomass which differed among groups (Fig. S3).

Taking into account separately the mixing and stratification periods, biomasses of dinoflagellates and ciliates were negatively correlated with depth during both periods (*P* < 0.05 and *P* < 0.001, Table S2); whereas the biomass of heterotrophic bacteria was negatively correlated with depth only during the mixing period (*P* < 0.05, Table S2). On the other hand, a negative correlation with depth was found for *Synechococcus* (*P* < 0.001), whereas a positive one for *Prochlorococcus* (*P* < 0.01) during only the stratification period (Table S2).

### Correlation between abiotic factors and biomass of food web component/trophic modes

The autotrophic (Auto), mixotrophic (Mixo) and heterotrophic (Hetero) biomass of the entire food web (measured separately in each group and summed up) was in the range of 1.18 (December, 120 m)–267.78 (June, 2 m), 0.02 (December, 75 m)–685.24 (July, 2 m) and 3.95 (December, 120 m)–93.30 (September, 75 m) μg C L^−1^, respectively (Table II). Mixotrophic biomass showed a significant positive correlation with temperature (*P* < 0.001) and a significant negative one with DIN (*P* < 0.05). Heterotrophic biomass did not show any significant correlation with any abiotic factor, whereas autotrophic biomass showed a significant positive correlation with temperature (*P* < 0.01, Table S3).

Mixotrophic biomass, instead, was positively correlated to temperature (*P* < 0.001) and negatively correlated to DIN (*P* < 0.05, Table S3).

More specifically, regarding different plankton groups, biomass of heterotrophic bacteria and diatoms did not show any significant correlation to abiotic variables or Chl a, whereas picoeukaryotes showed negative, significant correlations with temperature and salinity and positive, significant ones with PO_4_, DIN, SiO_4_ and Chl a (temperature: *P* < 0.05 and all the others: *P* < 0.01; Table S4). *Synechococcus* was significantly correlated only to temperature (*P* < 0.05), whereas *Prochlorococcus* was correlated to all variables except for PO_4_. More specifically, *Prochlorococcus* biomass was negatively correlated to temperature and salinity and positively correlated to nutrients and Chl a (Table S4). Both pigmented and nonpigmented nanoflagellate biomass showed a negative, significant correlation with temperature (*P* < 0.001 for both) and a positive, significant one with Chl a (*P* < 0.001 and *P* < 0.05, respectively). Moreover, pigmented nanoflagellates showed a positive, significant correlation also with DIN and SiO_4_ (*P* < 0.05 for both). Dinoflagellates and ciliates showed a positive correlation with temperature and a negative one with DIN (all *P* < 0.01; Table S4).

**Table II:** Range of biomass values for autotrophic, mixotrophic and heterotrophic components of the food web (μg C l^−1^) during each month

Month	Autotrophic		Mixotrophic		Heterotrophic	
	Min	Max	Min	Max	Min	Max
January	28.32	50.79	2.11	50.29	18.64	46.11
March	19.11	48.13	1.16	134.03	7.40	30.27
April	6.28	34.87	2.98	32.65	5.60	19.49
May	18.43	76.98	0.92	234.20	19.86	50.07
June	11.46	267.78	2.78	98.76	9.05	42.40
July	25.01	91.29	3.81	685.24	10.58	26.75
September	4.96	110.17	1.01	96.37	7.86	93.30
October	5.44	67.78	0.94	168.60	7.14	39.47
November	13.89	60.83	1.19	151.26	6.91	19.77
December	1.81	11.04	0.02	120.91	3.95	12.61

### Temporal and vertical distribution of pico-, nano- and microplankton biomass

**Fig. 3:**
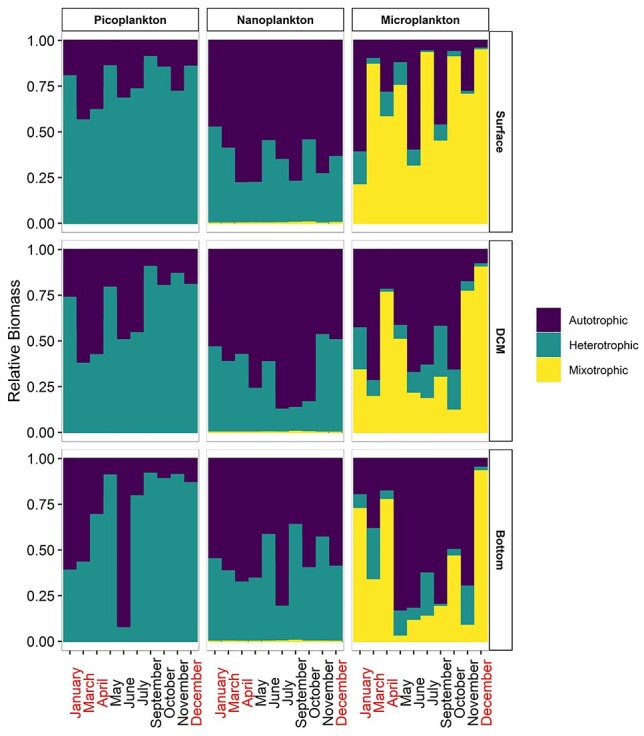
Distribution of autotrophic, mixotrophic and heterotrophic biomass in pico-, nano- and microplankton for each sample (all months surface, DCM and bottom layers). Months where the mixed water column occurred are inred.

The picoplankton fraction (heterotrophic bacteria, Cyanobacteria and picoeukaryotes) of the food web was mostly heterotrophic at the surface, DCM and bottom layers during the whole investigation period, except for the bottom layer of June, in which autotrophic biomass dominated (Fig. 3). The most pronounced heterotrophic conditions were in November and October due to the minimum values of Cyanobacteria abundance found. The nanoplankton fraction (pigmented and not pigmented flagellates, nanodinoflagellates and nanociliates) was mainly autotrophic in almost all cases except for June and September, at the bottom layers (Fig. 3). The microplankton fraction (dinoflagellates, diatoms and ciliates) was mostly mixotrophic (on average 41% ± 35). The highest micromixotrophic biomass was detected at the surface for almost all months and it decreased at DCM and bottom, except for January and December when mixotrophic biomass increased even further and remained constant, respectively, with depth (Fig. 3). The monthly range of the ratio of integrated Hetero:Auto biomass was 0.14–5.21, 0.18–0.95 and 0.15–0.55 for picoplankton, nanoplankton and microplankton, respectively, whereas regarding annual average at different depths, the ratio of Hetero:Auto biomass was 0.90–1.74, 0.48–0.78 and 0.19–0.49, respectively (Table III). The lowest values in terms of annual ratio average were found at 100 m for pico- and microplankton and at 20 m for nanoplankton, whereas the highest ones were at 2, 10 and 75 m, respectively, for pico-, nano- and microplankton.

**Table III:** Minimum and maximum values of the ratio between heterotrophic and autotrophic (Hetero:Auto), mixotrophic and heterotrophic (Mixo:Hetero) and mixotrophic and autotrophic (Mixo:Auto) biomass for pico-, nano- and microplankton

		Picoplankton			Nanoplankton			Microplankton		
Integrated	Month	Hetero:Auto	Mixo:Hetero	Mixo:Auto	Hetero:Auto	Mixo:Hetero	Mixo:Auto	Hetero:Auto	Mixo:Hetero	Mixo:Auto
	January	1.22	NA	NA	0.90	0.001	2.41	0.47	3.20	1.50
	March	0.49	NA	NA	0.67	0.001	1.12	0.23	4.22	0.96
	April	0.57	NA	NA	0.63	0.001	1.49	0.23	14.57	3.28
	May	2.44	NA	NA	0.34	0.001	1.54	0.23	5.52	1.26
	June	0.14	NA	NA	0.73	0.002	2.40	0.15	2.75	0.41
	July	0.86	NA	NA	0.18	0.004	4.64	0.28	5.32	1.48
	September	5.21	NA	NA	0.26	0.020	13.62	0.55	1.44	0.79
	October	2.48	NA	NA	0.29	0.009	5.91	0.31	3.04	0.94
	November	2.73	NA	NA	0.95	0.000	0.04	0.22	9.42	2.05
	December	2.28	NA	NA	0.87	0.002	0.50	0.18	77.01	13.65
Annual average	Depth									
	2	1.74	NA	NA	0.49	0.004	0.03	0.23	9.82	2.30
	10	1.57	NA	NA	0.78	0.002	0.02	0.20	40.29	8.24
	20	1.66	NA	NA	0.48	0.004	0.04	0.27	3.92	1.04
	50	1.34	NA	NA	0.58	0.002	0.02	0.24	2.42	0.58
	75	1.24	NA	NA	0.57	0.002	0.02	0.49	1.97	0.97
	100	0.90	NA	NA	0.72	0.001	0.01	0.19	2.66	0.50
	120	1.15	NA	NA	0.74	0.001	0.01	0.37	1.45	0.54

NA: not applicable

For both pico- and microplankton, the lowest values of integrated Hetero:Auto ratio was found in June and the highest one in September, whereas for nanoplankton, the lowest value was in July and the highest one in November. In terms of integrated and annual average of Mixo:Hetero biomass ratios, values were very low for nanoplankton, ranging between 0.0001–0.02 and 0.001–0.004, respectively. In microplankton, instead, integrated Mixo:Hetero and Mixo:Auto biomass ratios ranged between 1.44–77.01 and 0.41–13.65, respectively; whereas annual average ratios ranged between 1.45 and 40.29, and 0.50 and 8.24, respectively (Table III).

### Ciliates: Temporal and vertical distribution of trophic modes and size classes

The entire dataset comprised 30 677 ciliate specimens. From this number, a total of 21 462 (~71% of total) ciliate specimens were assigned to species level. From the rest 29%, a total of 3776 specimens were assigned to the genus *Pelagostrobilidium* and 202 to the genus *Strombidinopsis.* Species belonging to these two genera are known to be part of microplankton and also they belong to the order Choreotrichida. For this reason, they were all assigned to HmCil group. The rest 1555 specimens (5% of total ciliate specimens) were assigned to the genus *Strombidium* but further assignment to the species level was not possible. For this reason, they were not considered in our analyses. According to Romano *et al.* (2021)), the average distribution of the entire ciliate community to MnCil, HnCil, MmCil and HmCil based on epifluorescence counts was 5, 19, 20 and 57%, respectively, whereas this distribution when based on samples fixed with Lugol, was 6, 24, 31 and 38% for MnCil, HnCil, MmCil and HmCil, respectively.

Integrated values of MnCil were 0.16–7.66 × 10^6^ cells m^−2^ for abundance, and 0.04–3.48 × 10^6^ μg C m^−2^ for biomass; values for MmCil were 4.88–23.29 × 10^6^ cells m^−2^ for abundance and 5.74–33.35 × 10^6^ μg C m^−2^ for biomass (Table IV). Regarding heterotrophic ciliates, for nano- and microciliates abundance was 6.39–16.77 and 6.03–23.94 × 10^6^ cells m^−2^ and biomass 0.59–2.33 and 11.45–54.42 × 10^6^ μg C m^−2^, respectively (Table IV).

**Table IV:** Integrated abundance (10^3^ cells m^−2^) and integrated biomass (10^6^ μg C m^−2^) of mixotrophic nano- and microciliates (MnCil, MmCil) and heterotrophic nano- and microciliates (HnCil, HmCil)

	Abundance				Biomass			
Month	MnCil	HnCil	MmCil	HmCil	MnCil	HnCil	MmCil	HmCil
January	7.66	9.81	12.51	11.09	2.18	1.53	18.14	17.51
March	2.27	13.26	9.71	8.37	0.75	1.72	10.69	11.81
April	2.75	12.47	19.77	6.03	0.64	1.68	17.83	11.45
May	2.15	9.28	11.55	12.99	0.53	1.53	23.16	25.66
June	3.94	8.74	21.72	18.11	1.76	1.39	33.35	38.70
July	2.94	16.77	10.00	17.35	0.83	2.33	14.55	25.27
September	4.80	9.73	23.29	23.94	3.48	1.50	33.21	54.42
October	4.56	6.39	12.93	19.11	1.71	0.71	26.16	33.68
November	0.16	6.74	4.88	11.54	0.04	0.95	7.16	20.86
December	0.90	6.58	7.03	13.65	0.26	0.59	5.74	17.81

**Fig. 4:**
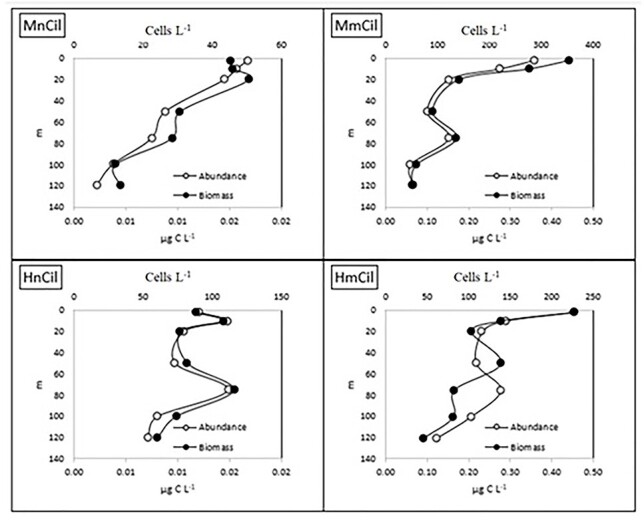
Annual average of abundance and biomass of MnCil, MmCil, HnCil and HmCil.

In terms of vertical distribution, the annual average of abundance and biomass of nanociliates at each depth showed a different pattern depending on trophic strategy. Mixotrophic nanociliate numerical abundance and biomass decreased significantly with depth (*P* < 0.05, data not shown), whereas heterotrophic nanociliates’ abundance and biomass were higher at 10 and 75 m depth (Fig. 4). Regarding microciliates on the other hand, both mixotrophs and heterotrophs showed the highest abundance and biomass at the surface and both decreased until DCM, where the second highest values were detected (Fig. 4).

Regarding nanociliate biomass, a significant positive correlation was found only between heterotrophic species and SiO_4_, whereas mixotrophic nanociliates did not show any significant correlation with any abiotic factor (Table V). As for microciliates, heterotrophic and mixotrophic biomass was positively correlated with temperature and negatively correlated with DIN (also with PO_4_ in the case of heterotrophic biomass, Table V).

**Table V:** Correlation between biomass of mixotrophic nano- and microciliates, and heterotrophic nano- and microciliates with abiotic factors

	HnCIL		MnCIL		HmCIL		MmCIL	
	*R*	*P*	*R*	*P*	*R*	*P*	*R*	*P*
Temperature	−0.05	Ns	0.20	NS	**0.64**	0.00^***^	**0.31**	0.02^*^
Salinity	0.08	Ns	0.15	NS	0.25	NS	0.19	NS
DIN	−0.09	Ns	−0.23	NS	**−0.47**	0.00^***^	**−0.37**	0.00^***^
PO_4_	−0.03	Ns	−0.15	NS	**−0.28**	0.03^*^	−0.13	NS
SIO_4_	**0.28**	0.03^*^	0.07	NS	−0.16	NS	−0.14	NS
Chl a	0.20	NS	−0.12	NS	−0.19	NS	−0.08	NS

Significant values are in bold.

NS, not significant.

^*^P < 0.05, ^**^P < 0.01, ^***^P < 0.001

### Biodiversity analysis of planktonic ciliates

**Fig. 5:**
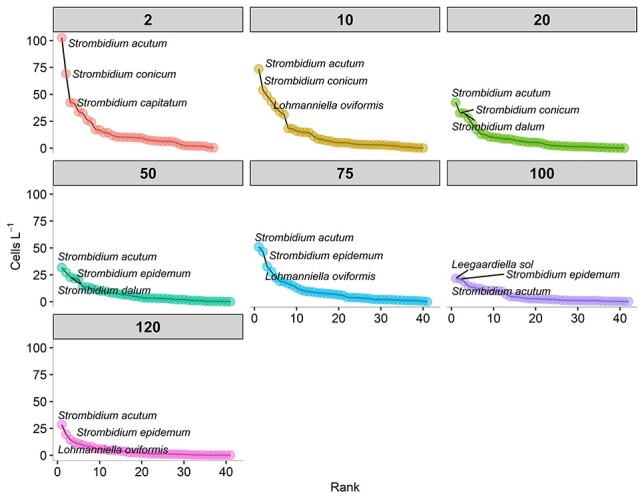
Rank abundance curve of annual average of abundance of pelagic ciliates at each depth.

Rank abundance curves showed that the most abundant ciliate species throughout the study belonged to the *Strombidium* genus, except for 100 m where the most abundant genus was *Leegaardiella* (Fig. 5). *Strombidium acutum* and *Strombidium conicum,* two mixotrophic micro-sized ciliates, were the most abundant species at the surface of the water column, specifically at 2, 10 and 20 m depth (Fig. 5); these two species contributed for most of the MmCil biomass at the surface layer. *S. acutum* and *Strombidium epidemum* were dominant species at the deeper layers, specifically at 50, 75 and 120 m depth; *S. epidemum* contributed for the most of HnCil biomass at 75 m (Fig. 5).

**Fig. 6:**
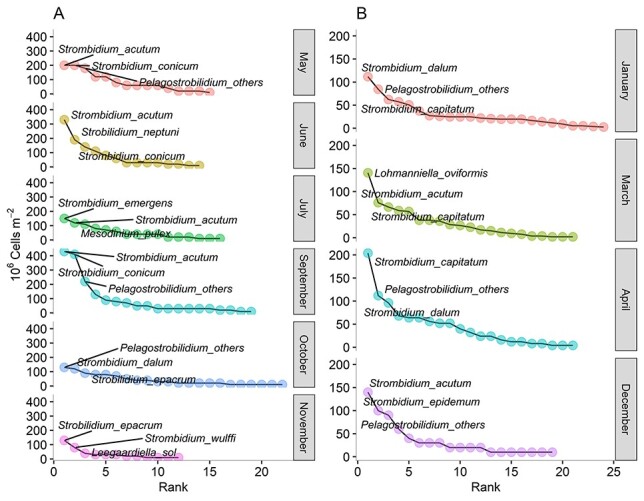
Rank abundance analysis of pelagic ciliate species during stratified (**A**) and mixed (**B**) water.

Rank abundance analysis, conducted on numerical abundance of planktonic ciliate species separately for the mixing and stratification periods, showed that most of the samples belonging to the mixing period (Fig. 6A) were mostly very diverse and no clear dominance was detected. During the stratification period (Fig. 6B), in the June and September samples, some species were more abundant compared to the others. More specifically, *S. acutum* and *S. conicum* abundances were two times higher compared to other species’ abundances (Fig. 6B). *Strombidium* was the most dominant genus in all months except for March and October when the genera dominating the ciliate community were *Lohmanniella* and *Strobilidium,* respectively.

Shannon index H, species richness and Pielou’s eveness were calculated for the integrated abundance of pelagic ciliates at each month sampled (Fig. S4) and also for the annual average at each depth (Fig. S4). The biodiversity analysis conducted at each month showed the highest Shannon index in September, whereas the lowest values were found in April, November and December (Fig. S4). Values for the other months ranged between 2.8 and 3.0. January showed the highest ciliate species richness value but the lowest Evenness value. *Vice versa* for October and May that showed very low values of species richness but high values of Evenness.

Regarding the annual average at each depth (Fig. S5), Shannon’s H index value was in the same range except for 50 and 120 m that showed the highest and the lowest values, respectively. Species richness increased exponentially between 2 and 20 m, whereas it remained stable down to 120 m, with the highest value found at 100 m. On the other hand, Evenness values decreased with depth, except for 2 and 50 m that showed the highest values. More specifically, those indices showed that the surface was populated by few species with homogenous distribution, but at the bottom, the number of species increased and so did also the dominance of some species compared to the others (Fig. S5).

### Correlation of different ciliate groups and their potential prey

**Fig. 7:**
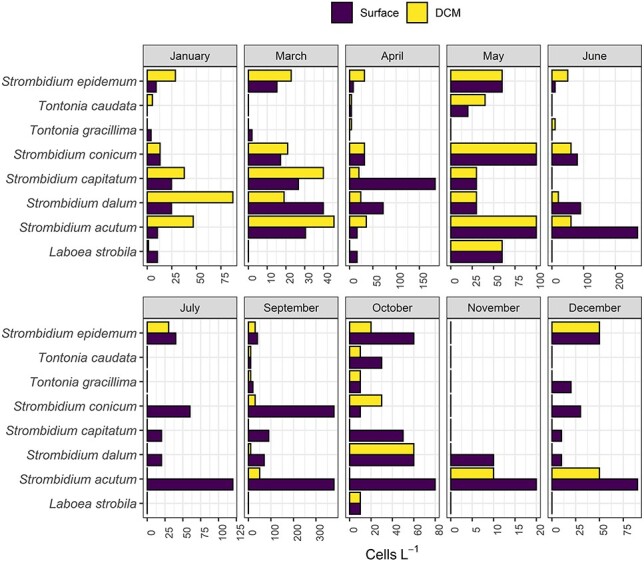
Distribution of the most abundant ciliate species at each month for surface andDCM.

The temporal distribution at the surface and DCM, of the most abundant ciliate species for the four functional groups showed that the summer months (June, July and September) were characterized by the dominance of two mixotrophic species: *S. acutum* and *S. conicum*; the abundance of these species was higher in the surface compared to the DCM, whereas during January, March and April, both *S. acutum* and *S. conicum* were more abundant at DCM compared to the surface (Fig. 7).

Pearson correlation conducted between abundances of ciliates and their potential prey showed that, at the surface layer, only the nanociliate species *Strombidium dalum* was negatively correlated to *Synechococcus* (*P* < 0.05). At the DCM layer, more ciliate species showed correlations with their potential prey; *S. dalum* showed a positive correlation with PnFlag (*P* < 0.05), whereas *L. strobila* and *Tontonia caudata* were positively correlated to *Synechoccoccus* (*P* < 0.01), finally *Strombidium capitatum* showed strong correlations with NPnFlag, PnFlag, picoeukaryotes and *Prochlorococcus* (*P* < 0.05 and *P* < 0.001, Table VI).

The CCA, run for the surface layers (Fig. S6A), showed that mixotrophic species such as *L. strobila* and *T. caudata* were grouped together with heterotrophic bacteria and *Synechococcus*, whereas *S. dalum* was grouped with PnFlag. On the other hand, at DCM *T. caudata* was grouped together with *Synechococcus*, whereas *S. capitatum* was very close to *Prochlorococcus* (Fig. S6B)*.*

## DISCUSSION

Our investigation, based on samplings in 10 months and 7 depths at a coastal station in the Eastern Mediterranean Sea, is a complete dataset of the temporal and vertical dynamics of the MFW components in an ultra-oligotrophic environment. Our results are at first a significant contribution to the knowledge of the MFW dynamics and pelagic ciliate biodiversity. Moreover, our results provide an insight on the relationships between pelagic ciliates and their potentialprey.

Abiotic and biotic variables measured in the present study were found similar to earlier studies (Pitta and Giannakourou, 2000; Siokou-Frangou *et al.,* 2010; Techtmann *et al.,* 2015). Low Chl a concentration were measured in all months and depths sampled, reflecting the ultra-oligotrophic status of the Eastern Mediterranean Sea. The coldest months of the mixing period, more specifically March, were characterized by high Chl a values and high concentration of PO_4_ and DIN, especially at 10 m depth. These results are supported by records from earlier studies conducted in the Eastern Mediterranean Sea, where the highest nutrient concentrations were detected especially during March and April (Azov, 1991; Bojanić *et al.,* 2001; Krom *et al.,* 2010; Moutin and Raimbault, 2002). In our study, this increase in Chl a, and nutrients during the months of March and April appeared together with the bloom of diatoms. On the other hand, during the hottest months, such as July, the lowest Chl a concentration was recorded in all depth layers. This anomaly probably allowed the growth of heterotrophic and mixotrophic protists such as heterotrophic dinoflagellates and mixotrophic ciliates during summer (June to September).

**Table VI:** Pearson correlation coefficient with P values of some species of GNCMs and their potential prey at surface andDCM

Depth	Predator	Prey	*R*	*P*
Surface	*Strombidium dalum*	*Synechococcus*	−0.66	0.04^*^
DCM	*Tontonia caudata*	*Synechococcus*	0.85	0.00^***^
	*Laboea strobila*	*Synechococcus*	0.86	0.00^***^
	*Strombidium dalum*	HnFlag	0.74	0.01^**^

NPnFlag: ^*^P < 0.05, ^**^P < 0.01, ^***^P < 0.001.

### Pico-, nano- and microplankton

The components of the MFW showed different temporal and vertical dynamics. Pico- and nanoplankton were the most abundant groups and were present in all depths and months sampled, like in most oligotrophic areas (Brunet *et al.,* 2008; Casotti *et al.,* 2003; Dolan *et al.,* 2002; Ignatiades *et al.,* 2002; Tanaka *et al.,* 2007; Yacobi *et al.,* 1995). In the present study, the components <20 μm, commonly defined as nanoplankton, were mainly constituted not only of small flagellates (generally < 10 μm) but also of dinoflagellates, mostly naked species, in addition to a limited number of very small ciliate species (Pitta and Giannakourou, 2000; Siokou-Frangou *et al.,* 2010). The analysis of the food web, considered as a whole, showed the low abundances and dominance of nanoplanktonic groups, especially during the cold months. Moreover, pigmented nanoflagellates were correlated with DIN showing their dependence on inorganic nutrients (Arenovski *et al.,* 1995; Christaki *et al.,* 1999; Tsai *et al.,* 2011).

In contrast to nanoplankton that showed a clear temporal trend dominating in the cold, mixing period as well as a significant decrease with depth, picoplankton did not show any temporal or vertical distribution pattern but dominated the plankton biomass during the entire study period and in all depths. In terms of microplankton, the highest abundance and biomass were recorded during the warm, stratification period.

In previous records from the South Aegean Sea, the contribution of pico-, nano- and microplankton to the whole pelagic community was 22, 61 and 17%, respectively (Ignatiades *et al.,* 2002; Sammartino *et al.,* 2015; Vidussi *et al.,* 2001). These studies were focused mostly on short time periods (only spring, or only one month with a focus on spatial dynamics). Furthermore, other studies reported only on autotrophic and heterotrophic biomass, without considering the mixotrophic one (Aytan *et al*., 2017); for this reason, it is very difficult to compare our results with previous records from the Eastern Mediterranean Sea. The annual contribution of pico-, nano- and microplankton to total microbial biomass, in our study, ranged between 8.01–20.01%, 26.47–43.16% and 37.33–65.51%, respectively (Table S5, Fig. S7). The contribution of picoplankton to total biomass increased with depth, and the biomass of heterotrophic bacteria was higher below the DCM in most of the months, together with the increase of nutrients in the bottom compared to the surface. This supports the idea that heterotrophic bacteria are strictly related to nutrient availability, especially in oligotrophic systems (Christaki *et al.,* 1999). Nanoplankton’s contribution, instead, remained constant throughout depths, whereas microplankton biomass was higher above the DCM, compared to the bottom layers (75, 100 and 120 m). Microplankton was mostly dominated by dinoflagellates and most of the dinoflagellate species are considered to be autotrophic or mixotrophic. These two specific trophic modes depend on light to perform photosynthesis which fact may explain their position in the higher layers of the water column.

### Autotrophy, mixotrophy and heterotrophy

Mixotrophy may be conceptualized as a strategy that allows organisms to compete with heterotrophs for prey and with autotrophs for nutrients and light (Edwards, 2019). Irradiance may also play an important role in mixotrophic dynamics through its variability with depth and time/month. Because they may obtain carbon through photosynthesis, mixotrophic organisms can survive also in environments where there is prey scarcity. In our study, annual average contribution of autotrophic, mixotrophic and heterotrophic to total microbial biomass fell in the ranges 28.01–53%, 9.44–53.55% and 18.44–41.38%, respectively; the highest percentages were found at DCM (50 and 75 m), 10 m and bottom layer, respectively. The lowest percentage of both autotrophic and heterotrophic biomasses was found at 10 m depth, where the highest values of nutrients and potential prey abundances were encountered. In the present study, mixotrophic biomass was higher at 10 m depth, where dissolved nutrients are higher, and during summer, where light availability and prey are relative abundant but nutrients are scarce. So, for this reason, it is possible that mixotrophic organisms are more related to light compared to nutrients. This result is similar to other records from oligotrophic environments, such as, the Atlantic Ocean (Barton *et al.,* 2013), where autotrophic biomass (dominated by diatoms) was prevalent during spring, and mixotrophic biomass (dominated by dinoflagellates) was prevalent during summer (Barton *et al.,* 2013).

Moreover, it is known that oligotrophic waters are dominated by heterotrophic picoplankton biomass (Aytan *et al.,* 2017; Fuhrman *et al.,* 1989; González-Benítez *et al.,* 2019). In our study, we showed that the degree of dominance by heterotrophic biomass may change with time but not with depth. In all depths heterotrophic bacteria dominated; however, if we take into account the seasonal variability, the observed system switched between autotrophic biomass (dominated by nanoflagellates) during January–March to heterotrophic biomass (dominated by heterotrophic bacteria) during October–January. On the other hand, the mixotrophic biomass (represented by micro- dinoflagellates and ciliates) could be seen as the “intermediate” state of the environment, between photo-autotrophy and heterotrophy. In other words, in oligotrophic waters which are dominated by pico- and nanoplankton, the heterotrophic picoplankton biomass increases during the most productive periods of nutrient excess, and it is in turn controlled by grazing exerted by nanoflagellates, which are mostly pigmented (autotrophic and mixotrophic) (Fuhrman *et al.,* 1989). However, the mixotrophic microplankton biomass that increases during the stratification period, in theory may control both heterotrophic and autotrophic biomass since mixotrophic microplanktonic organisms might compete with heterotrophs for prey and with autotrophs for nutrients.

### Ciliate abundance and diversity

Concerning the species composition of microplankton, dinoflagellates were the most important group in terms of species number and biomass. They characterized all months and depths, even if their higher contribution, in terms of cell abundance, was observed during the warmer months.

In terms of ciliate diversity, this coastal station at the Eastern Mediterranean was found different from the Western basin. The pelagic ciliate community in the oligotrophic Eastern Mediterranean was diverse and 47 species belonging to 22 genera and 3 orders were identified during 1-year sampling. As far as aloricate ciliates are concerned though, it is not possible to compare the species number with other studies because of taxonomical uncertainties due to fixation methods. Both Lugol and formaldehyde may affect the numerical abundance and the shape of cells. For this reason, it is strongly recommended to count two parallel series of samples, one fixed with Lugol and another one with formaldehyde in order to assess the cell loss (best achieved in Lugol fixed samples) but also consider mixotrophy prevailing in this group of organisms (possible to study only in formaldehyde fixed samples) (Karayanni *et al.,* 2004).

Regarding tintinnids, a total of 22 species were identified in our study. This number is lower compared to other studies conducted in the Mediterranean Sea, such as in Adriatic waters, where a total of 38 tintinnid species were identified (Bojanić *et al.,* 2001; Heneash *et al.,* 2015; Polat *et al.,* 2019). Aloricate ciliates constitute the majority of the ciliate community (Dolan and Marrasé, 1995; Ferrier-Pages and Rassoulzadegan, 1994). The highest density of these organisms in the Mediterranean Sea (~39 000 cells L^−1^) was recorded by Revelante and Gilmartin (1983). In our study, the majority of aloricates were found in the surface layers down to 20 m depth, as described in many earlier studies (Bojanić *et al.,* 2001; Bojanić *et al.,* 2005; Pitta and Giannakourou, 2000; Rekik *et al.,* 2021).

The analysis of the ciliate community composition at all months and depths sampled revealed the distinction of the stratified vs mixed water column, in terms of temporal variability on one hand and of the surface vs DCM layers, in terms of vertical variability on the other. Taking into account the ciliate community in relation with the other components of the MFW, such as dinoflagellates (potential competitors) and pigmented nanoflagellates (potential prey), the CCA analysis showed that the same ciliate species may be connected with different potential prey according to depth. This finding may be interpreted as an indication of many different factors acting to define the ciliate potential impact on the other components of the MFW. These results seem to confirm that the ciliate community is extremely diverse and plastic according to environmental variables and most probably to the presence/absence of potential and specific prey).

### Potential correlation between ciliates and components of pico- and nanoplankton

The structure of the MFW was different between the surface and DCM depth layers and also between mixed (January, March, April and December), and stratified (May, June, July, September, October and November) periods. The big changes in temperature affected the dynamics of the MFW components and in turn this affected the distribution of different functional and size groups of ciliates. During summer (stratified water column), most of the components of the MFW were detected at the surface in higher densities compared to the DCM and this confinement was probably due to the occurrence of the thermocline. Because of the big difference in temperature between the surface and DCM, the recycling of nutrients was very scarce and this may have affected the pico- and nanoplankton dynamics. As a consequence, most ciliate species that were abundant at the DCM at the mixed water column could be detected only at the surface layers during June, July and September.

The most abundant ciliate species belonged to the *Strombidium* genus. This result is comparable to other studies conducted in the Eastern Mediterranean Sea (Bojanić *et al.,* 2001; Pitta and Giannakourou, 2000; Romano *et al.,* 2021).

If we look at the correlations between large and small ciliates with the other components of the food web, taking also into account the ciliate trophic modes, we may detect and disentangle the different dynamics prevailing in different ciliate size classes as previously reported in Romano *et al.* (2021). Pearson correlations and CCA revealed different dynamics of the ciliate community at the surface and DCM, depending on ciliate size and trophic mode. More specifically, large mixotrophic ciliate species, like *L. strobila* and *S. capitatum*, showed a correlation with *Synechococcus* and *Prochlorococcus,* respectively. Moreover, these ciliate species showed a correlation with the two cyanobacteria species only at the surface layer and not at DCM. This could be explained by the fact that large ciliate species (>50 μm) were abundant in the water column only above DCM and only in a certain period of the year. In contrast, the MnCil species *S. dalum* showed different correlations with different potential preys according to the depth layer analyzed. At the surface, *S. dalum* was correlated with *Synechococcus*, whereas at DCM the correlation was significant with pigmented nanoflagellates. In Romano *et al.* (2021), large and small mixotrophic species were shown to be differently distributed throughout the water column and this result could support the hypothesis that large mixotrophic species are more selective in terms of potential prey, whereas small species could be more generalist and be correlated with different kind of prey according to season and depth. It is difficult, though, to prove this hypothesis only based on 1-year sampling at one station; however, our dataset could represent an important starting point for further studies in the field and also in cultures.

On the other hand, *S. conicum* and *S. acutum* were very abundant during summer months (June, July and September), especially at the surface. Both *S. conicum* and *S. acutum* are mixotrophic species belonging to microplankton (> 20 μm).

Rank abundance curves showed that those species were the most dominant ones during June and September at 2 m depth. DIN and temperature were the most important factors for the dynamics of *S. conicum* and *S. acutum*. A positive correlation with temperature and a negative one with DIN may indicate a potential connection between these species with the warmer part of theyear.

Pigmented and nonpigmented nanoflagellates may be another food source for ciliates (Solic and Krstulovic, 1994), as well as heterotrophic bacteria and Cyanobacteria (Sherr and Sherr, 1987). During the warmer months, total nanoflagellates were very low and this could be an indication of potential grazing exerted by ciliates onthem.

## CONCLUSIONS

The goal of the present study was to investigate the temporal and vertical dynamics of different size groups of mixotrophic and heterotrophic ciliates and their correlation with the other components of the MFW. Biomasses of different components of the MFW showed different temporal and vertical variability, except for heterotrophic bacteria that dominated in all months and depths independently from any abiotic or biotic factor. Moreover, picoplankton and nanoplankton were dominated by heterotrophic and autotrophic biomasses, respectively, and their biomasses were more important during cold months. Mixotrophic biomass was more important for the microplankton, comprising most of the ciliate species, and microplankton biomass was, on the other hand, more important during summer. In other words, it is clear that picoplankton dominated all months and depths, but nanoplankton could take advantage of the nutrients availability in winter, leaving space to microplankton in summer. Furthermore, mixotrophy took advantage in the period of the year where nutrients are scarce. The impact of mixotrophic ciliates on their potential prey differed with time and depth. More specifically, large species like *L. strobila* were correlated with Cyanobacteria only at DCM, but smaller mixotrophic species like *S. dalum* were correlated with different components of the MFW according to depth. More specifically, the nanociliate *S. dalum* was correlated with *Synechococcus* at the surface and with nonpigmented nanoflagellates at DCM. This result reflects the fact that larger species could potentially be more selective in terms of prey, whereas smaller ciliates are more generalists. Furthermore, it is possible that, in unstable environments, such as oligotrophic marine systems, mixotrophic ciliates could be driven by different abiotic and potential prey factors.

## Supplementary Material

supplementary_paper_II_fbab053

## Data Availability

The original contributions presented in the study are included in the manuscript/Supplementary material, further inquiries can be directed to the corresponding author/s.

## References

[ref1] Agawin, N. S. R., Duarte, C. M. and Agustí, S. (1998) Growth and abundance of Synechococcus sp. in a Mediterranean Bay: seasonality and relationship with temperature. Mar. Ecol. Prog. Ser., 170, 45–53. 10.3354/meps170045.

[ref2] Arenovski, A. L., Lim, E. L. and Caron, D. A. (1995) Mixotrophic nanoplankton in oligotrophic surface waters of the Sargasso Sea may employ phagotrophy to obtain major nutrients. J. Plankton Res., 17, 801–820. 10.1093/plankt/17.4.801.

[ref3] Aytan, U., Feyzioglu, A. M., Valente, A., Agirbas, E. and Fileman, E. S. (2017) Microbial plankton communities in the coastal southeastern Black Sea: biomass, composition and trophic interactions. Oceanologia., 60, 139–152. 10.1016/j.oceano.2017.09.002.

[ref4] Azam, F., Fenchel, T., Field, J., Gray, J., Meyer-Reil, L. and Thingstad, F. (1983) The ecological role of water-column microbes in the sea. Mar. Ecol. Prog. Ser., 10, 257–263. 10.3354/meps010257.

[ref5] Azov, Y. (1991) Eastern Mediterranean-a marine desert? Mar. Pollut. Bull., 23, 225–232. 10.1016/0025-326X(91)90679-M.

[ref6] Barton, A. D., Finkel, Z. V., Ward, B. A., Johns, D. G. and Follows, M. J. (2013) On the roles of cell size and trophic strategy in North Atlantic diatom and dinoflagellate communities. Limnol. Oceanogr., 58, 254–266. 10.4319/lo.2013.58.1.0254.

[ref7] Bojanić, N., Šolić, M., Krstulović, N., Marasović, I., Ninčević, Ž. and Vidjak, O. (2001) Seasonal and vertical distribution of the ciliated protozoa and micrometazoa in Kaštela Bay (central Adriatic). Helgol. Mar. Res., 55, 150–159. 10.1007/s101520000067.

[ref8] Bojanić, N., Šolić, M., Krstulović, N., Šestanović, S., Marasović, I. and Ninčević, Ž. (2005) Temporal variability in abundance and biomass of ciliates and copepods in the eutrophicated part of Kaštela Bay (Middle Adriatic Sea). Helgol. Mar. Res., 59, 107–120. 10.1007/s10152-004-0199-x.

[ref9] Bojanić, N., Vidjak, O., Šolić, M., Krstulović, N., Brautović, I., Matijević, S., Kušpilić, G., Šestanovi, S. et al. (2012) Community structure and seasonal dynamics of tintinnid ciliates in Katela Bay (middle Adriatic Sea). J. Plankton Res., 34, 510–530. 10.1093/plankt/fbs019.

[ref10] Brunet, C., Casotti, R. and Vantrepotte, V. (2008) Phytoplankton diel and vertical variability in photobiological responses at a coastal station in the Mediterranean Sea. J. Plankton Res., 30, 645–654. 10.1093/plankt/fbn028.

[ref11] Burkill, P. H., Edwards, E. S., John, A. W. G. and Sleigh, M. A. (1993) Microzooplankton and their herbivorous activity in the northeastern Atlantic Ocean. Deep-Sea Res. II, 40, 479–493. 10.1016/0967-0645(93)90028-L.

[ref12] Capriulo, G. M., Sherr, E. B. and Sherr, B. F. (1991) Trophic behaviour and related community feeding activities of heterotrophic marine Protists. Prot. and Their Role in Mar. Proc., 219–265. 10.1007/978-3-642-73181-5_16.

[ref13] Caron, D. A., Dam, H. G., Kremer, P., Lessard, E. J., Madin, L. P., Malone, T. C., Napp, J. M., Peele, E. R. et al. (1995) The contribution of microorganisms to particulate carbon and nitrogen in surface waters of the Sargasso Sea near Bermuda. Deep-Sea Res. I, 42, 943–972. 10.1016/0967-0637(95)00027-4.

[ref14] Caroppo, C. (2000) The contribution of picophytoplankton to community structure in a Mediterranean brackish environment. J. Plankton Res., 22, 381–397. 10.1093/plankt/22.2.381.

[ref15] Caroppo, C., Turicchia, S. and Margheri, M. C. (2006) Phytoplankton assemblages in coastal waters of the northern Ionian Sea (eastern Mediterranean), with special reference to cyanobacteria. J. Mar. Biol. Assoc. UK, 86, 927–937. 10.1017/S0025315406013889.

[ref16] Casotti, R., Landolfi, A., Brunet, C., D’Ortenzio, F., Mangoni, O., Ribera d’Alcalà, M. and Denis, M. (2003) Composition and dynamics of the phytoplankton of the Ionian Sea (eastern Mediterranean). J. Geophys. Res. Oceans, 108. 10.1029/2002jc001541.

[ref17] Christaki, U., Van Wambeke, F. and Dolan, J. R. (1999) Nanoflagellates (mixotrophs, heterotrophs and autotrophs) in the oligotrophic eastern Mediterranean: standing stocks, bacterivory and relationships with bacterial production. Mar. Ecol. Prog. Ser., 181, 297–307.

[ref18] Dolan, J. R. and Marrasé, C. (1995) Planktonic ciliate distribution relative to a deep chlorophyll maximum: Catalan Sea, NW Mediterranean, June 1993. Deep-Sea Res., 42, 1965–1987. 10.1016/0967-0637(95)00092-5.

[ref19] Dolan, J. R., Claustre, H., Carlotti, F., Plounevez, S. and Moutin, T. (2002) Microzooplankton diversity: relationships of tintinnid ciliates with resources, competitors and predators from the Atlantic Coast of Morocco to the Eastern Mediterranean. Deep-Sea Res. I, 49, 1217–1232. 10.1016/S0967-0637(02)00021-3.

[ref20] Dolan, J. R., Ciobanu, M., Marro, S., Coppola, L. and Ji, R. (2019) An exploratory study of heterotrophic protists of the mesopelagic Mediterranean Sea. ICES J. Mar. Sci., 76, 616–625. 10.1093/icesjms/fsx218.

[ref21] Edwards, K. F. (2019) Mixotrophy in nanoflagellates across environmental gradients in the ocean. PNAS, 116. 10.1073/pnas.1814860116. PMC6442547 30760589

[ref22] El-Shabrawy, G. M., Anufriieva, E. V. and Shadrin, N. V. (2018) Tintinnina (Ciliophora) and foraminifera in plankton of hypersaline lagoon bardawil (Egypt): spatial and temporal variability. Turk. J. Zool., 42, 218–229. 10.3906/zoo-1705-37.

[ref23] Fenchel, T. (2008) The microbial loop - 25 years later. J. Exp. Mar. Biol. Ecol., 366, 99–103. 10.1016/j.jembe.2008.07.013.

[ref24] Ferrier-Pages, C. and Rassoulzadegan, F. (1994) Seasonal impact of the microzooplankton on pico- and nanoplankton growth rates in the Northwest Mediterranean Sea. Mar. Ecol. Prog. Ser., 108, 283–294. 10.3354/MEPS108283.

[ref25] Fuhrman, J. A., Sleeter, T. D., Carlson, C. A. and Proctor, L. M. (1989) Dominance of bacterial biomass in the Sargasso Sea and its ecological implications. Mar. Ecol. Prog. Ser., 57, 2017–2017. 10.3354/meps057207.

[ref26] Fuhrman, J. A. (1999) Marine viruses and their biogeochemical and ecological effects. Nature, 399, 541–548. 10.1038/21119. 10376593

[ref27] Garrison, D. L., Gowing, M. M., Hughes, M. P., Campbell, L., Caron, D. A., Dennett, M. R., Shalapyonok, A., Olson, R. J. et al. (2000) Microbial food web structure in the Arabian Sea: a US JGOFS study. Deep-Sea Res. II, 47, 1387–1422. 10.1016/S0967-0645(99)00148-4.

[ref28] Gasol, J. M., Del Giorgio, P. A. and Duarte, C. M. (1997) Biomass distribution in marine planktonic communities. Limnol. Oceanogr., 42, 1353–1363. 10.4319/lo.1997.42.6.1353.

[ref29] González-Benítez, N., García-Corral, L. S., Morán, X. A. G., Middelburg, J. J., Pizay, M. D. and Gattuso, J. P. (2019) Drivers of microbial carbon fluxes variability in two oligotrophic Mediterranean coastal systems. Sci. Rep., 9, 17669. 10.1038/s41598-019-53650-z. 31776462 PMC6881365

[ref30] Hall, J. A. and Vincent, W. F. (1994) Vertical and horizontal structure of the picophytoplankton community in a stratified coastal system off New Zealand. N. Z. J. Mar. Freshwater Res., 28, 299–308. 10.1080/00288330.1994.9516617.

[ref31] Halse, G. R., Syvertsen, E. E., Steidinger, K. A., and Tangen, K. (1996). Identifying Marine Diatoms and Dinoflagellates. 10.1016/B978-0-12-693015-3.X5000-1

[ref32] Heneash, A. M. M., Abdel-Rahman, N. S. and Gharib, S. M. (2015) Community composition, abundance and biomass of tintinnids (ciliata: Protozoa) in the Western Harbour, South-Eastern Mediterranean Sea, Egypt. Environ. Monit. Assess., 187. 10.1007/s10661-015-4735-8. 26202815

[ref33] Ignatiades, L., Psarra, S., Zervakis, V., Pagou, K., Souvermezoglou, E., Assimakopoulou, G. and Gotsis-Skretas, O. (2002) Phytoplankton size-based dynamics in the Aegean Sea (Eastern Mediterranean). J. Mar. Syst., 36, 11–28. 10.1016/S0924-7963(02)00132-X.

[ref34] Ivančič, I. and Degobbis, D. (1984) An optimal manual procedure for ammonia analysis in natural waters by the indophenol blue method. Water Res., 18, 1143–1147. 10.1016/0043-1354(84)90230-6.

[ref35] Karayanni, H., Christaki, U., Van Wambeke, F. and Dalby, A. P. (2004) Evaluation of double formalin - Lugol’s fixation in assessing number and biomass of ciliates: an example of estimations at mesoscale in NE Atlantic. J. Microbiol. Methods, 56, 349–358. 10.1016/j.mimet.2003.11.002. 14967226

[ref36] Krom, M. D., Emeis, K. C. and Van Cappellen, P. (2010) Why is the Eastern Mediterranean phosphorus limited? Prog. Oceanogr., 85, 236–244. 10.1016/j.pocean.2010.03.003.

[ref37a] Laval-Peuto, M. and Rassoulzadegan, F. (1988) Autofluorescence of marine planktonic Oligotrichina and other ciliates. Hydrobiologia, 159, 99–110. doi: 10.1007/BF00007371.

[ref37] Le Bescot, N., Mahé, F., Audic, S., Dimier, C., Garet, M. J., Poulain, J., Wincker, P., de Vargas, C. et al. (2016) Global patterns of pelagic dinoflagellate diversity across protist size classes unveiled by metabarcoding. Environ. Microbiol., 18, 609–626. 10.1111/1462-2920.13039. 26337598

[ref38] Lee, S. and Fuhrman, J. A. (1987) Relationships between biovolume and biomass of naturally derived marine bacterioplankton. Appl. Environ. Microbiol., 53, 1298–1303. 10.1128/aem.53.6.1298-1303.1987. 16347362 PMC203858

[ref39] Li, W. K. W., Dickie, P. M., Irwin, B. D. and Wood, A. M. (1992) Biomass of bacteria, cyanobacteria, prochlorophytes and photosynthetic eukaryotes in the Sargasso Sea. Deep Sea Res. I, 39, 501–519. 10.1016/0198-0149(92)90085-8.

[ref39a] Lynn, D. H., Montagnes, D. J. S., Dale, T., Gilron, G. L. and Strom, S. L. (1991) A reassessment of the genus strombidinopsis (ciliophora, choreotrichida) with descriptions of four new planktonic species and remarks on its taxonomy and phylogeny. J. Mar. Biol. Assoc. U.K., 71, 597–612. doi: 10.1017/S0025315400053170.

[ref40] Otero-Ferrer, J. L., Cermeño, P., Bode, A., Fernández-Castro, B., Gasol, J. M., Morán, X. A. G., Marañon, E., Moreira-Coello, V. et al. (2018) Factors controlling the community structure of picoplankton in contrasting marine environments. Biogeosciences, 15, 6199–6220. 10.5194/bg-15-6199-2018.

[ref41] Mackey, D. J., Blanchot, J., Higgins, H. W. and Neveux, J. (2002) Phytoplankton abundances and community structure in the equatorial Pacific. Deep-Sea Res. II, 49, 2561–2582. 10.1016/S0967-0645(02)00048-6.

[ref42] Marie, D., Brussaard, C. P. D., Thyrhaug, R., Bratbak, G. and Vaulot, D. (1999) Enumeration of marine viruses in culture and natural samples by flow cytometry. Appl. Environ. Microbiol., 65, 45–52. 10.1128/aem.65.1.45-52.1999. 9872758 PMC90981

[ref43] Matishov, G., Makarevich, P., Timofeev, S., Kuznetsov, L., Druzhkov, N., Larionov, V., Golubev, V., Zuyev, A. et al. (2000) Biological atlas of the Arctic Seas 2000: plankton of the Barents and Kara Seas. International Ocean Atlas Series, 2, 348 hdl:10013/epic.42580.d025.

[ref44] Menden-Deuer, S. and Lessard, E. J. (2000) Carbon to volume relationships for dinoflagellates, diatoms, and other protist plankton. Limnol. Oceanogr., 45, 569–579. 10.4319/lo.2000.45.3.0569.

[ref45] Modigh, M., Saggiomo, V. and Ribera D’Alcalà, M. (1996) Conservative features of picoplankton in a Mediterranean eutrophic area, the Bay of Naples. J. Plankton Res., 18, 87–95. 10.1093/plankt/18.1.87.

[ref46a] Montagnes, D. J. S., Lynn, D. H., Stoecker, D. K. and Small, E. B. (1988) Taxonomic descriptions of one new species and Redescription of four species in the Family Strombidiidae (Ciliophora, Oligotrichida). J. Protozool., 35, 189–197. doi: 10.1111/j.1550-7408.1988.tb04322.x.

[ref46] Moutin, T. and Raimbault, P. (2002) Primary production, carbon export and nutrients availability in Western and Eastern Mediterranean Sea in early summer 1996 (MINOS cruise). J. Mar. Syst., 33-34, 273–288. 10.1016/S0924-7963(02)00062-3.

[ref47] Partensky, F., Blanchot, J., and Vaulot, D. (1999). Differential distribution and ecology of Prochlorococcus and Synechococcus in oceanic waters: a review. Bulletin-Institut Oceanographique Monaco-Numero Special-, 457–476.

[ref48] Pitta, P. and Giannakourou, A. (2000) Planktonic ciliates in the oligotrophic Eastern Mediterranean: vertical, spatial distribution and mixotrophy. Mar. Ecol. Prog. Ser., 194, 269–282. 10.3354/meps194269.

[ref49] Polat, S., Terbiyik Kurt, T. and Tugrul, S. (2019) Spatial and temporal variations of tintinnids (Ciliata: Protozoa) in the Bay of Mersin, Northeastern Mediterranean Sea. Mediterr. Mar. Sci., 20, 342–356. 10.12681/mms.18074.

[ref50] Pomeroy, L. R. (1974) The ocean’s food web, a changing paradigm. Bioscience, 24, 499–504. 10.2307/1296885.

[ref51] Porter, K. G. and Feig, Y. S. (1980) The use of DAPI for identifying and counting aquatic microflora. Limnol. Oceanogr., 25, 943–948. 10.4319/lo.1980.25.5.0943.

[ref52] Putt, M. and Stoecker, D. K. (1989) An experimentally determined carbon: volume ratio for marine “oligotrichous” ciliates from estuarine and coastal waters. Limnol. Oceanogr., 34, 1097–1103. 10.4319/lo.1989.34.6.1097.

[ref53] Revelante, N. and Gilmartin, M. (1983) Microzooplankton distribution in the Northern Adriatic Sea with emphasis on the relative abundance of ciliated protozoans. Oceanol. Acta, 6, 407–415.

[ref54] Rekik, A., Kmiha-Megdiche, S., Drira, Z., Pagano, M., Ayadi, H., Bellaaj Zouari, A. and Elloumi, J. (2021) Spatial variations of planktonic ciliates, predator-prey interactions and their environmental drivers in the Gulf of Gabes-Boughrara lagoon system. Estuar. Coast. Shelf Sci., 254, 107315. 10.1016/j.ecss.2021.107315.

[ref55] Rimmelin, P. and Moutin, T. (2005) Re-examination of the MAGIC method to determine low orthophosphate concentration in seawater. Anal. Chim. Acta, 548, 174–182. 10.1016/j.aca.2005.05.071.

[ref56] Romano, F., Symiakaki, K. and Pitta, P. (2021) Temporal variability of planktonic ciliates in a coastal oligotrophic environment: mixotrophy, size classes and vertical distribution. Front. Mar. Sci., 8, 1–14. 10.3389/fmars.2021.641589.

[ref57] Sammartino, M., Di Cicco, A., Marullo, S. and Santoleri, R. (2015) Spatio-temporal variability of micro-, nano- and pico-phytoplankton in the Mediterranean Sea from satellite ocean colour data of SeaWiFS. Open Sci., 11, 759–778. 10.5194/os-11-759-2015.

[ref58] Sanders, R. W., Caron, D. A. and Berninger, U. G. (1992) Relationships between bacteria and heterotrophic nanoplankton in marine and fresh waters: an inter-ecosystem comparison. Mar. Ecol. Prog. Ser., 86, 1–14. 10.3354/meps086001.

[ref59] Sherr, E. B. and Sherr, B. F. (1987) High rates of consumption of bacteria by pelagic ciliates. Nature, 325, 710–711. 10.1038/325710a0.

[ref60] Sherr, E. B. and Sherr, B. F. (1988) Role of microbes in pelagic food webs: a revised concept. Limnol. Oceanogr., 33, 1225–1227. 10.4319/lo.1988.33.5.1225.

[ref61] Siokou-Frangou, I., Christaki, U., Mazzocchi, M. G., Montresor, M., Ribera D’Alcala, M., Vaque, D. and Zingone, A. (2010) Plankton in the open Mediterranean Sea: a review. Biogeosciences, 7, 1543–1586. 10.5194/bg-7-1543-2010.

[ref62] Solic, M. and Krstulovic, N. (1994) Role of predation in controlling bacterial and heterotrophic nanoflagellate standing stocks in the coastal Adriatic Sea: seasonal patterns. Mar. Ecol. Prog. Ser., 114, 219–235. 10.3354/meps114219.

[ref63] Strickland, J. D. H. and Parsons, T. R. (1972) A Practical Handbook of Seawater Analysis. In J. Fish. Res. Board Can, 167, p. 310.

[ref64] Tanaka, T., Zohary, T., Krom, M. D., Law, C. S., Pitta, P., Psarra, S., Rassoulzadegan, F., Thingstad, T. F. et al. (2007) Microbial community structure and function in the Levantine Basin of the eastern Mediterranean. Deep-Sea Res. I, 54, 1721–1743. 10.1016/j.dsr.2007.06.008.

[ref65] Techtmann, S. M., Fortney, J. L., Ayers, K. A., Joyner, D. C., Linley, T. D., Pfiffner, S. M. and Hazen, T. C. (2015) The unique chemistry of Eastern Mediterranean water masses selects for distinct microbial communities by depth. PLoS One, 10, 1–22. 10.1371/journal.pone.0120605. PMC4373936 25807542

[ref66] Trombetta, T., Vidussi, F., Roques, C., Scotti, M. and Mostajir, B. (2020) Marine microbial food web networks during phytoplankton bloom and non-bloom periods: warming Favors smaller organism interactions and intensifies trophic Cascade. Front. Microbiol., 11, 1–19. 10.3389/fmicb.2020.502336. 33193116 PMC7644461

[ref67] Tsai, A. Y., Gong, G. C., Sanders, R. W., Chen, W. H., Chao, C. F. and Chiang, K. P. (2011) Importance of bacterivory by pigmented and heterotrophic nanoflagellates during the warm season in a subtropical western Pacific coastal ecosystem. Aquat. Microb. Ecol., 63, 9–18. 10.3354/ame01470.

[ref68] Utermöhl, H. (1931). Neue Wege in der quantitativen Erfassung des Plankton.(Mit besonderer Berücksichtigung des Ultraplanktons.). SIL Proceedings, 1922-2010. 10.1080/03680770.1931.11898492.

[ref69] Van Dongen-Vogels, V., Seymour, J. R., Middleton, J. F., Mitchell, J. G. and Seuront, L. (2012) Shifts in picophytoplankton community structure influenced by changing upwelling conditions. Estuar. Coast. Shelf Sci., 109, 81–90. 10.1016/j.ecss.2012.05.026.

[ref70] Verity, P. G. and Langdon, C. (1984) Relationships between lorica volume, carbon, nitrogen, and ATP content of tintinnids in Narragansett Bay. J. Plankton Res., 6, 859–868. https://doi.org/10.1093/ plankt/6.5.859.

[ref71] Vidussi, F., Claustre, H., Manca, B. B., Luchetta, A. and Marty, J. C. (2001) Phytoplankton pigment distribution in relation to upper thermocline circulation in the Eastern Mediterranean Sea during winter. J. Geophys. Res.: Oceans, 106, 19939–19956. 10.1029/1999jc000308.

[ref72] Whitman, W. B., Coleman, D. C. and Wiebe, W. J. (1998) Prokaryotes: the unseen majority. PNAS, 95, 6578–6583. 10.1073/pnas.95.12.6578. 9618454 PMC33863

[ref73] Yacobi, Y. Z., Zohary, T., Kress, N., Hecht, A., Robarts, R. D., Waiser, M., Wood, A. M. and Li, W. K. W. (1995) Chlorophyll distribution throughout the southeastern Mediterranean in relation to the physical structure of the water mass. J. Mar. Syst., 6, 179–190. 10.1016/0924-7963(94)00028-A.

[ref74] Yang, J., Chen, Z., Chen, D. and Xu, D. (2021) Spatial distribution of the microzooplankton communities in the northern South China Sea: insights into their function in microbial food webs. Mar. Pollut. Bull., 162, 111898. 10.1016/j.marpolbul.2020.111898. 33316704

[ref75] Yentsch, C. S. and Menzel, D. W. (1963) A method for the determination of phytoplankton chlorophyll and phaeophytin by fluorescence. Deep-Sea Res., 10, 221–231. 10.1016/0011-7471(63)90358-9.

[ref76] Zubkov, M. V., Sleigh, M. A. and Burkill, P. H. (2000) Assaying picoplankton distribution by flow cytometry of underway samples collected along a meridional transect across the Atlantic Ocean. Aquat. Microb. Ecol., 21, 13–20. 10.3354/ame021013.

[ref77] Zubkov, M. V., Sleigh, M. A., Tarran, G. A., Burkill, P. H. and Leakey, R. J. G. (1998) Picoplanktonic community structure on an Atlantic transect from 50°N to 50°S. Deep-Sea Res. I, 45, 1339–1355. 10.1016/S0967-0637(98)00015-6.

