# Supplementary material for: Relationships of pelagic ciliates with the microbial food web components at a coastal station in the oligotrophic Eastern Mediterranean Sea: temporal and vertical variability
Source: J Plankton Res. 2021 Aug 27;43(5):691–711. doi: 10.1093/plankt/fbab053 (PMC8461642; doi:10.1093/plankt/fbab053)

**Table S1.** Value ranges of abiotic variables, nutrients and Chl a for each month. NA: not available.

|  | **Jan** | **Mar** | **Apr** | **May** | **Jun** | **Jul** | **Sep** | **Oct** | **Nov** | **Dec** |
| --- | --- | --- | --- | --- | --- | --- | --- | --- | --- | --- |
| **Temp**  °C | 16.14- 16.19 | 15.34- 15.87 | 16.03- 16.75 | 16.02- 19.26 | NA | 16.21- 26.59 | 16.66- 25.16 | 16.56- 22.82 | 16.71- 21.72 | 16.87- 20.40 |
| **Sal**  PSU | 39.24- 39.25 | 38.74- 39.21 | 39.25- 39.28 | 39.14- 39.34 | NA | 39.19- 39.35 | 39.00- 39.38 | 39.09- 39.31 | 39.08- 39.25 | 39.08- 39.26 |
| **DIN**  µM | 0.85 - 1.92 | 0.38 - 2.41 | 0.34 - 2.11 | 0.51 - 1.75 | NA | 0.32 – 1.14 | 0.49 - 1.34 | 0.49 - 1.19 | 0.62 - 0.98 | 0.47 - 1.19 |
| **PO_4_**  µM | 0.000- 0.003 | 0.01 - 0.090 | 0.000- 0.011 | 0.004- 0.011 | 0.001- 0.007 | 0.002 - 0.01 | 0.001- 0.006 | 0.002- 0.014 | 0.002- 0.013 | 0.001- 0.006 |
| **SiO_4_**  µM | 0.92 - 1.23 | 1.52 - 2.32 | 1.26 - 1.44 | 0.48 - 0.99 | NA | 1.13 – 2.09 | 1.00- 1.45 | 0.33 - 0.62 | 0.66 - 0.98 | 0.67 - 1.08 |
| **Chl a**  µg C L^-1^ | 0.18 - 0.22 | 0.00 - 0.79 | 0.09 - 0.32 | 0.07 - 0.38 | NA | 0.05 - 0.19 | 0.02 - 0.24 | 0.07 - 0.28 | 0.04 - 0.05 | 0.11 - 0.18 |

**Table S2.** Correlation between biomass of the microbial food web components and depth during the mixing and stratification periods. Significant p values are in bold, Ns = not significant. Bact = Heterotrophic bacteria; Syn = *Synechococcus*; Proc = Prochlorococcus; pEuk =picoeukaryotes;NPnFLAG = non-pigmented nanoflagellates; PnFLAG = pigmented nano-flagellates; Diat = Diatoms; Dino = Dinoflagellates; Cil = Ciliates. p < 0.05 *, p < 0.01 **, p < 0.001***.

|  | **Depth (mixed)** | | **Depth (stratified)** | |
| --- | --- | --- | --- | --- |
|  | R | p | R | p |
| **Bact** | **-0.38** | **0.03*** | -0.23 | ns |
| **Syn** | -0.21 | ns | **-0.66** | **0.00***** |
| **Proc** | 0.29 | ns | **0.46** | **0.01**** |
| **pEuk** | -0.31 | ns | 0.29 | ns |
| **NPnFlag** | -0.10 | ns | -0.10 | ns |
| **PnFlag** | -0.12 | ns | -0.30 | ns |
| **Dino** | **-0.41** | **0.02*** | **-0.53** | **0.00***** |
| **Diat** | 0.00 | ns | 0.01 | ns |
| **Cil** | **-0.50** | **0.00***** | **-0.52** | **0.00***** |

**Table S3.** Correlation between biomass of the autotrophic, mixotrophic and heterotrophic components of the plankton food web and abiotic factors. Significant p values are in bold, Ns = not significant. DIN: dissolved inorganic Nitrogen. p < 0.05 *, p < 0.01 **, p < 0.001***.

|  | ***Auto*** |  | ***Mixo*** |  | ***Het*** |  |
| --- | --- | --- | --- | --- | --- | --- |
|  | R | p | R | p | R | p |
| ***Temperature*** | **0.30** | **0.01**** | **0.48** | **0.00***** | -0.04 | ns |
| ***Salinity*** | 0.10 | ns | 0.20 | ns | -0.07 | ns |
| ***DIN*** | 0.03 | ns | **-0.28** | **0.02*** | 0.07 | ns |
| ***PO_4_*** | -0.05 | ns | -0.10 | ns | 0.00 | ns |
| ***SiO_4_*** | 0.18 | ns | -0.02 | ns | 0.12 | ns |
| ***Chla*** | 0.13 | ns | -0.11 | ns | 0.21 | ns |

**Table S4.** Correlation between biomass of the components of the microbial food web and abiotic factors and Chla. Ns = not significant. Bact = Heterotrophic bacteria; Syn = *Synechococcus*; Proc = *Prochlorococcus*; pEuk = picoeukaryotes; NPnFlag = Non-pigmented nanoflagellates; PnFlag = Pigmented nanoflagellates; Diat = Diatoms; Dino = Dinoflagellates; Cil = Ciliates; DIN: dissolved inorganic Nitrogen. p < 0.05 *, p < 0.01 **, p < 0.001***.

|  | **Temperature** | **Salinity** | **DIN** | **PO_4_** | **SIO_4_** | **Chla** |
| --- | --- | --- | --- | --- | --- | --- |
| **Bact** | ns | ns | ns | ns | ns | ns |
| **Syn** | **0.35*** | ns | ns | ns | ns | ns |
| **Proc** | **-0.56**** | **-0.30*** | **0.32*** | ns | **0.30*** | **0.44**** |
| **pEuk** | **-0.30*** | **-0.63**** | **0.57**** | **0.68**** | **0.59**** | **0.46**** |
| **NPnFlag** | **-0.40**** | ns | ns | ns | ns | **0.29*** |
| **PnFlag** | **-0.44**** | ns | **0.28*** | ns | **0.27*** | **0.38**** |
| **Dino** | **0.55**** | ns | **-0.37**** | ns | ns | ns |
| **Diat** | ns | ns | ns | ns | ns | ns |
| **Cil** | **0.51**** | ns | **-0.38**** | ns | ns | ns |

**Table S5.** Annual average of the relative biomass of pico, nano and microplankton at each depth.

| **Depth** | **Picoplankton** | **Nanoplankton** | **Microplankton** |
| --- | --- | --- | --- |
| **(m)** | % biomass | | |
| **2** | 10.13 | 29.11 | 60.76 |
| **10** | 8.01 | 26.47 | 65.51 |
| **20** | 10.16 | 31.50 | 58.34 |
| **50** | 10.98 | 39.17 | 49.84 |
| **75** | 16.21 | 41.59 | 42.20 |
| **100** | 19.51 | 43.16 | 37.33 |
| **120** | 20.01 | 41.40 | 38.59 |

**Fig. S1**. Area studied (ultra-oligotrophic Eastern Mediterranean Sea) with sampling location (coastal station Poseidon-HCB).


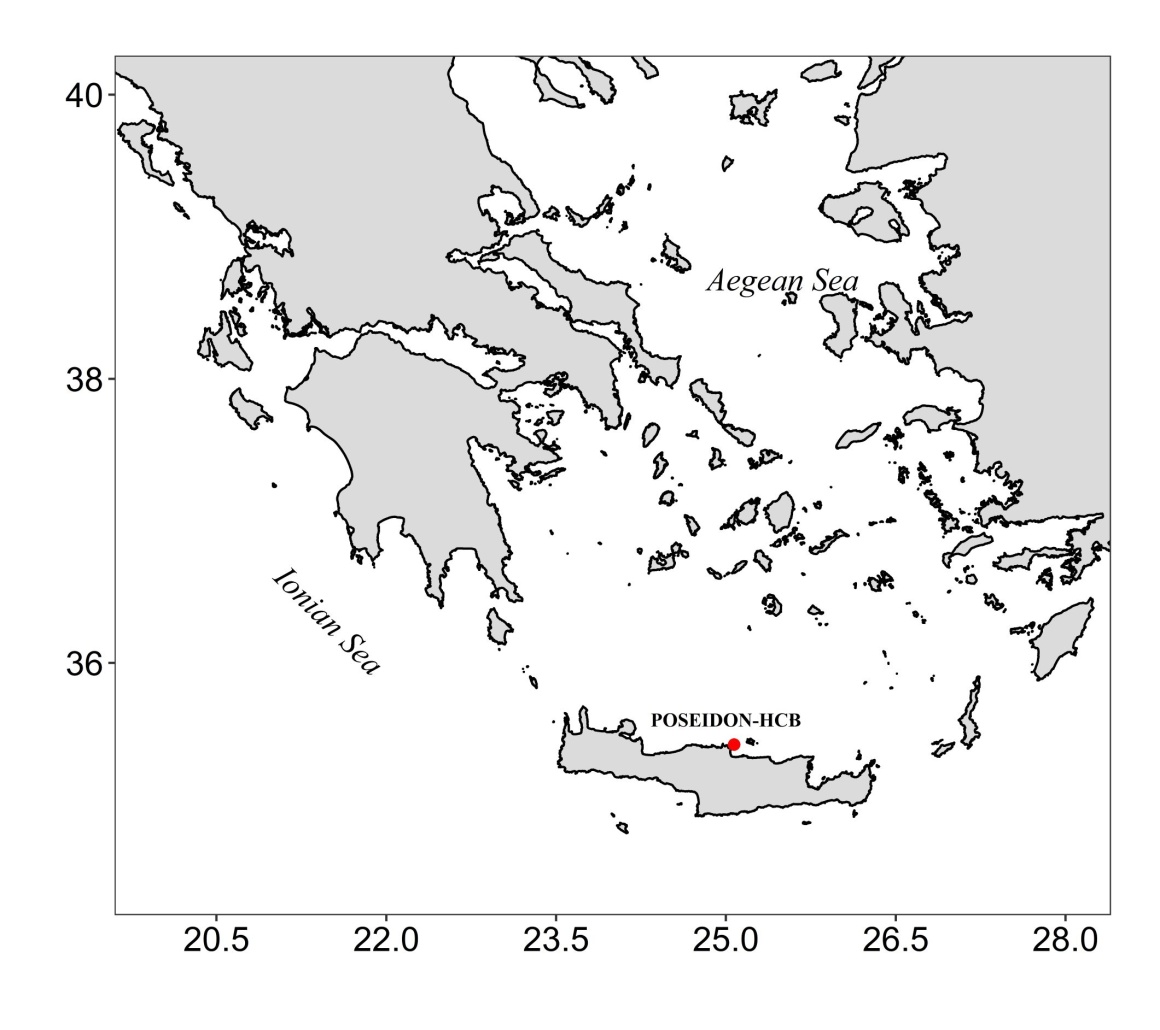


**Fig. S2** Vertical and temporal distribution of PO_4_ (A), DIN (B) and SiO_4_ (C) in HCB station during 2019.


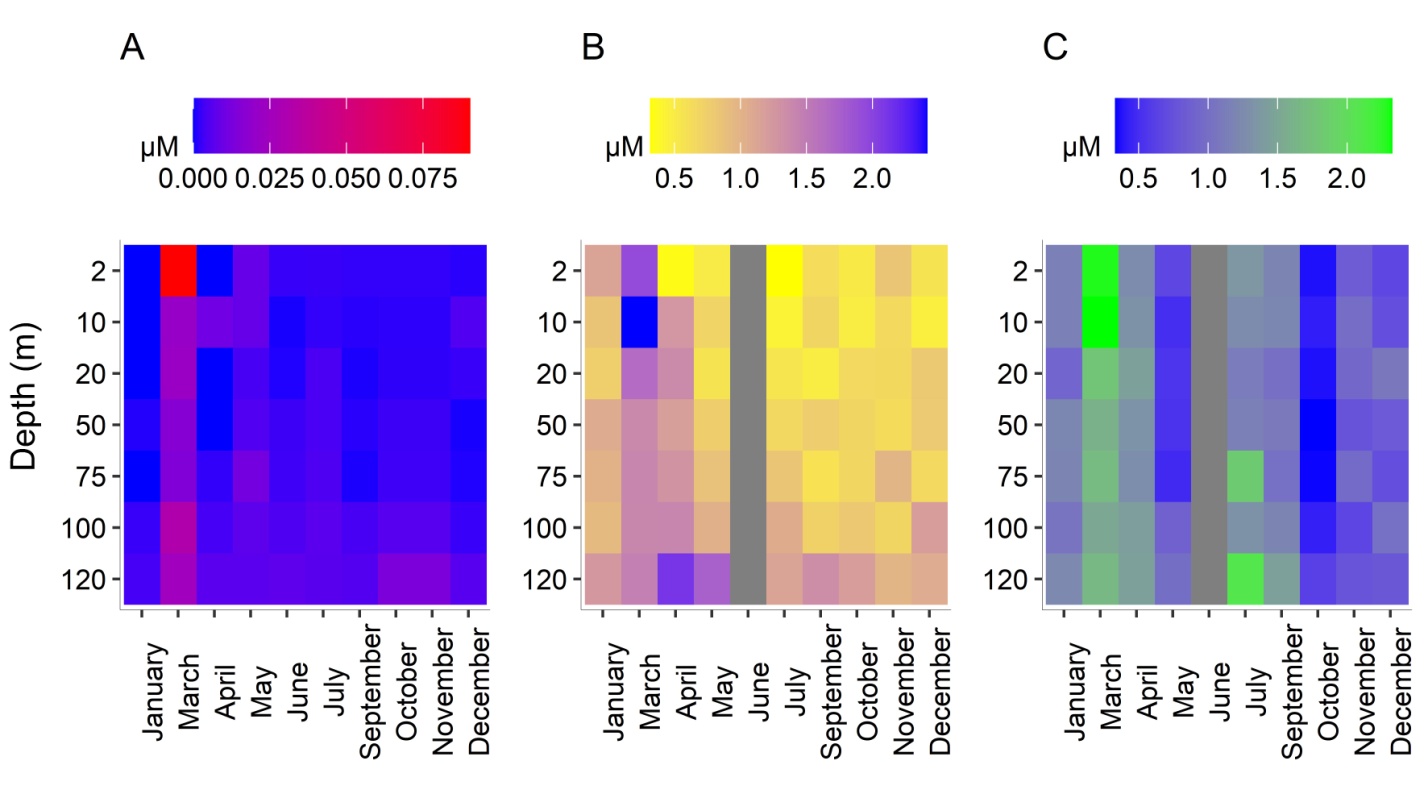


**Fig. S3** Annual average) of abundance and biomass of heterotrophic bacteria (A), *Synechococcus* (B), *Prochlorococcus* (C), Pigmented nanoflagellates (D), Non-pigmented nanoflagellates (E) picoeukaryotes (F), diatoms (G), dinoflagellates (H) and ciliates (I).Y axes represent depth (m), while the upper X axis represents abundance (cells L^-1^) and the lower X axis biomass (µg C L^-1^).


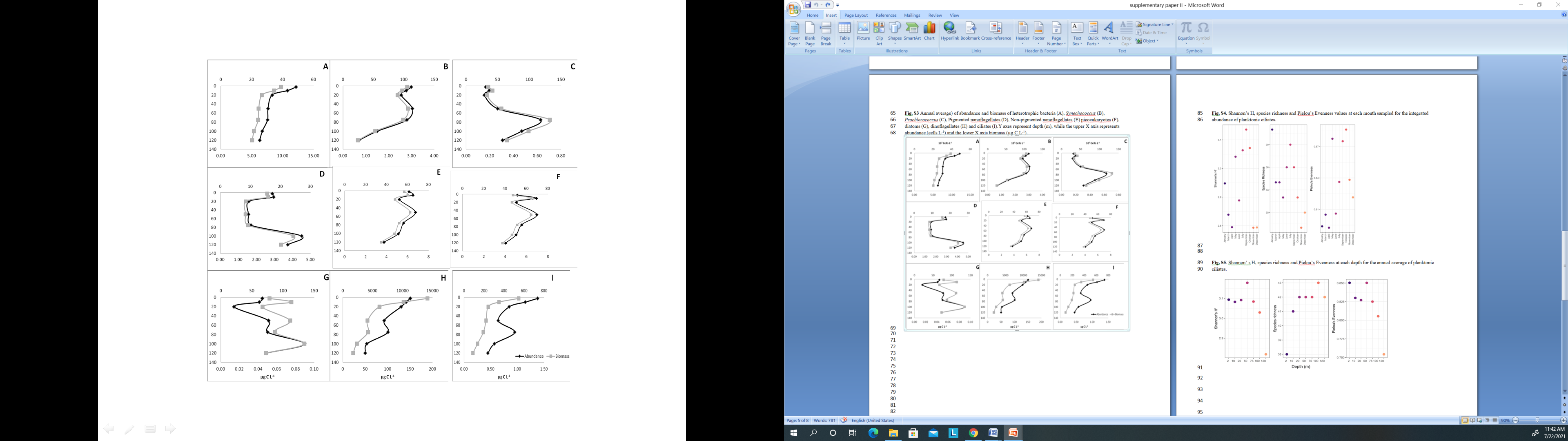


**Fig. S4.** Shannon’s H, species richness and Pielou’s Evenness values at each month sampled for the integrated abundance of planktonic ciliates.


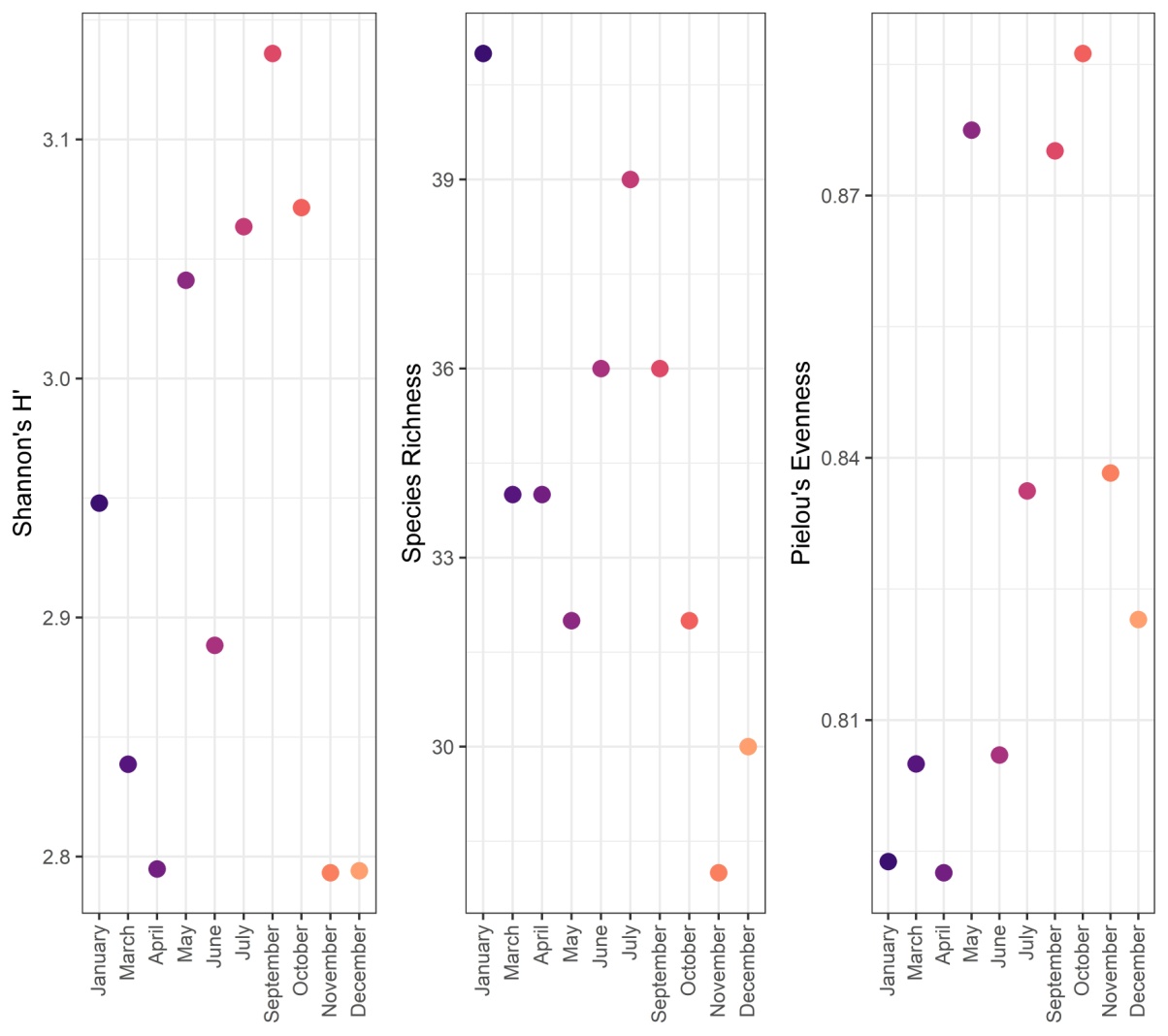


**Fig. S5**. Shannon’ s H, species richness and Pielou’s Evenness at each depth for the annual average of planktonic ciliates.


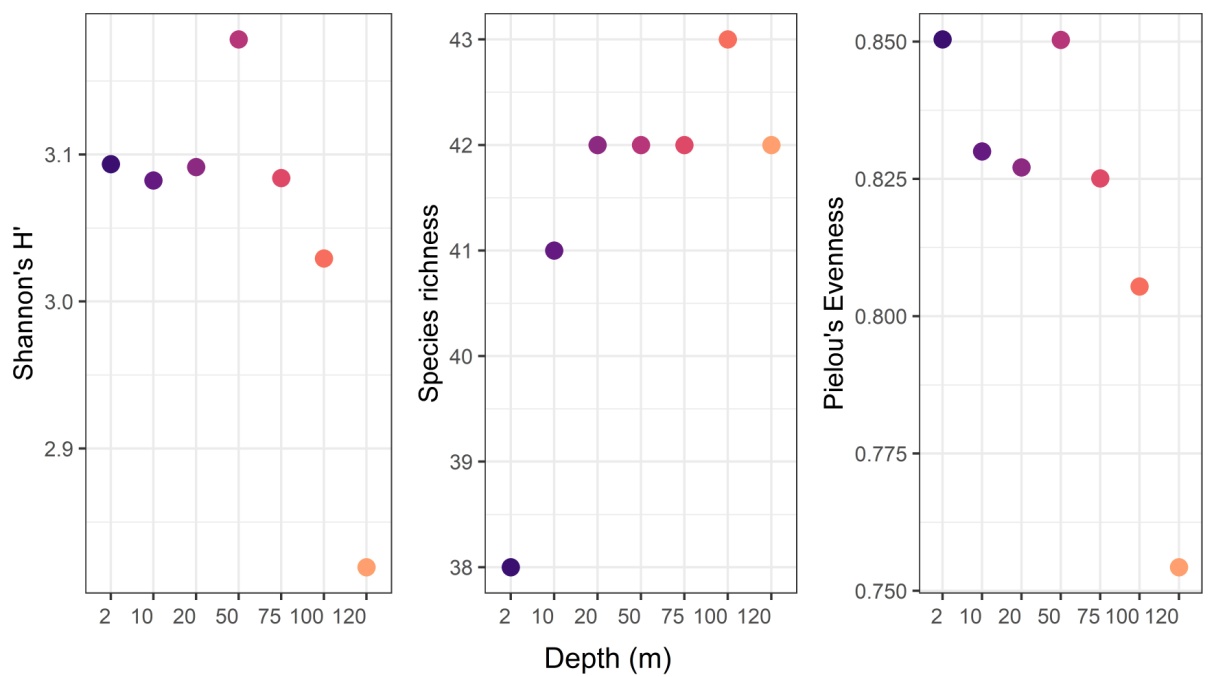


**Fig. S6**. Canonical correlation analysis (CCA) between ciliate species and their potential prey for both surface (A) and DCM (B).


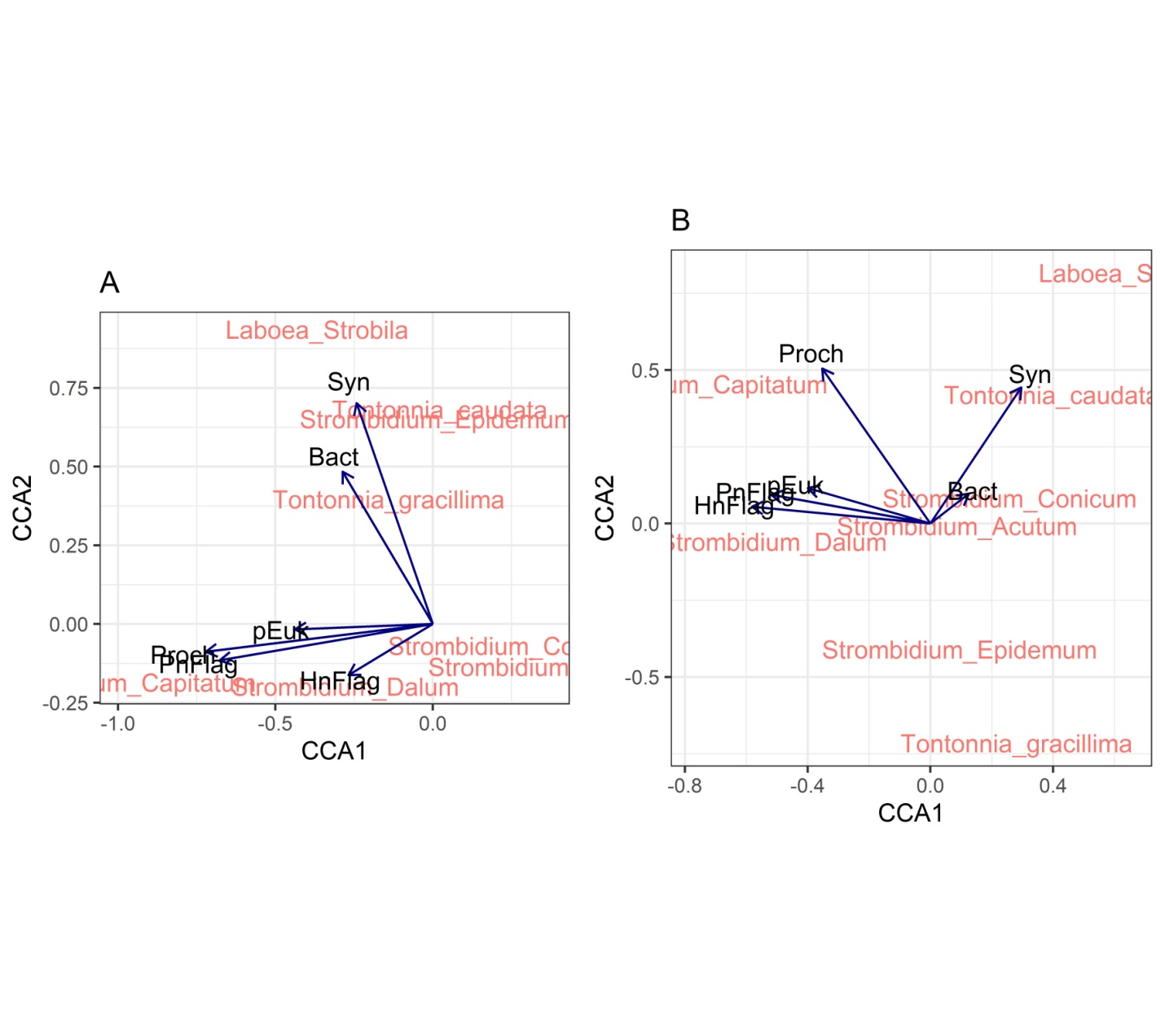


**Fig. S7**. Relative biomass at each depth (annual average) of autotrophic, mixotrophic and heterotrophic biomass (top) and picoplankton, nanoplankton and microplankton (bottom).


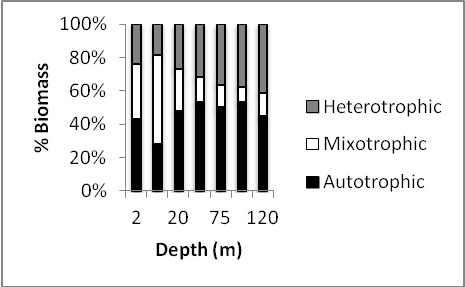


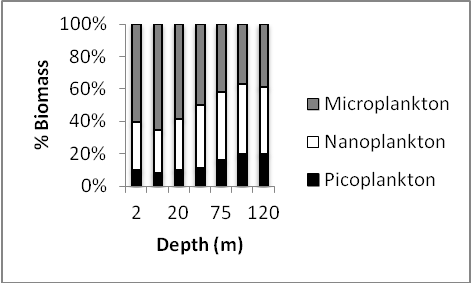


**Fig. S8** Scatter plot between Temperature and Density at each month sampled to check the state of the water column, either was stratified or mixed.


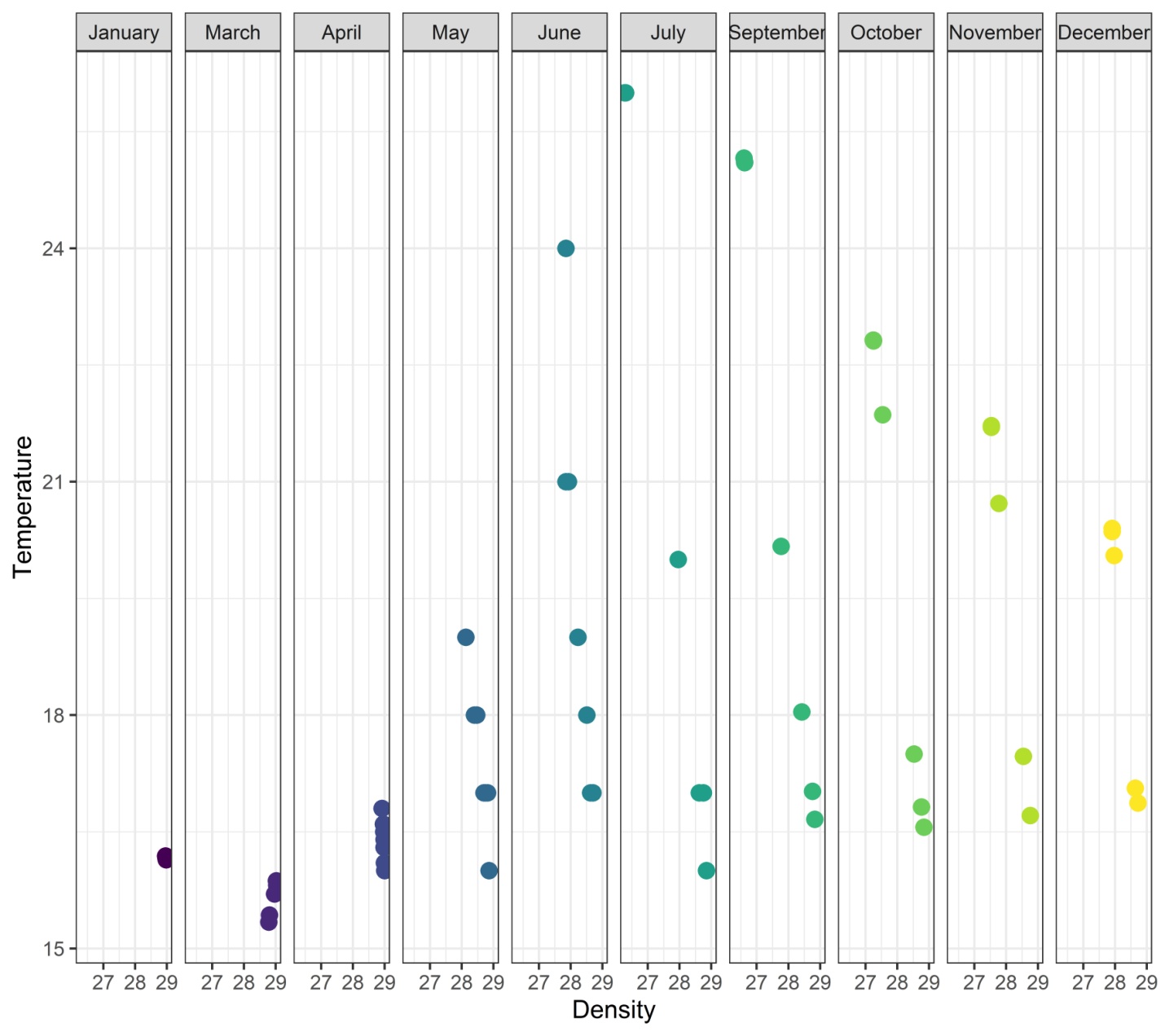

Supplement: supplementary_paper_II_fbab053 [file supplementary_paper_ii_fbab053.doc]
